# Divide to Conquer: Evolutionary History of Allioideae Tribes (Amaryllidaceae) Is Linked to Distinct Trends of Karyotype Evolution

**DOI:** 10.3389/fpls.2020.00320

**Published:** 2020-04-07

**Authors:** Lucas Costa, Horace Jimenez, Reginaldo Carvalho, Jefferson Carvalho-Sobrinho, Inelia Escobar, Gustavo Souza

**Affiliations:** ^1^Laboratory of Plant Cytogenetics and Evolution, Department of Botany, Federal University of Pernambuco, Recife, Brazil; ^2^Laboratory of Plant Cytogenetics, Department of Biology, Federal Rural University of Pernambuco, Recife, Brazil; ^3^Department of Botany, University of Concepción, Concepción, Chile

**Keywords:** Amaryllidaceae, BioGeoBEARS, biogeography, cytogenetics, rDNA sites, genome size, phylogenetic comparative methods (PCMs)

## Abstract

Allioideae (e.g., chives, garlics, onions) comprises three mainly temperate tribes: Allieae (800 species from the northern hemisphere), Gilliesieae (80 South American species), and Tulbaghieae (26 Southern African species). We reconstructed the phylogeny of Allioideae (190 species plus 257 species from Agapanthoideae and Amaryllidoideae) based on ITS, *mat*K, *ndh*F, and *rbc*L to investigate its historical biogeography and karyotype evolution using newly generated cytomolecular data for Chilean Gilliesieae genera *Gethyum*, *Miersia*, *Solaria*, and *Speea*. The crown group of Allioideae diversified ∼62 Mya supporting a Gondwanic origin for the subfamily and vicariance as the cause of the intercontinental disjunction of the tribes. Our results support the hypothesis of the Indian tectonic plate carrying Allieae to northern hemisphere (‘out-of-India’ hypothesis). The colonization of the northern hemisphere (∼30 Mya) is correlated with a higher diversification rate in *Allium* associated to stable *x* = 8, increase of polyploidy and the geographic expansion in Europe and North America. Tulbaghieae presented *x* = 6, but with numerical stability (2*n* = 12). In contrast, the tribe Gilliesieae (*x* = 6) varied considerably in genome size (associated with Robertsonian translocations), rDNA sites distribution and chromosome number. Our data indicate that evolutionary history of Allioideae tribes is linked to distinct trends of karyotype evolution.

## Introduction

The search for the causes of species geographic distributions is notable for its lack of universally applicable rules (Stebbins, 1971; Lowry and Lester, 2006; Araújo et al., 2019; Liu et al., 2019). The current species distribution reflects their dispersal ability, environmental tolerance, niche breadth, population abundance, colonization–extinction dynamics, and character diversity (Brown et al., 1996; Lowry and Lester, 2006). Geographic distribution may impact evolution; however, in the case of karyotype data (e.g., number and morphology of chromosomes, ploidy level correlated with genome size, number of ribosomal DNA [rDNA] sites, genome size) the opposite also occurs and chromosomal changes may lead to ability to colonize new environments (e.g., allopolyploids; Souza et al., 2012). In this context, cytogeography emerged as the analysis of the geographical distributions of polymorphic cytological markers, as polyploidy, inversions, Robertsonian translocations, increase/decrease of rDNA site number, etc. (Colombo and Confalonieri, 2004). Analyses of spatial distribution of karyotypes may indicate an adaptive value for certain types of chromosomal rearrangements and help to clarify processes that contributed to shape particular distribution patterns (Colombo and Confalonieri, 2004; Raskina et al., 2004; Van-Lume et al., 2017).

The classical cytogeographic analyses implemented by plotting cytotypes on maps now is furthered by incorporating modern approaches in a time-space interface by phylogenetic comparative methods (PCMs). Although PCMs are widely used in ecology (Legendre and Legendre, 2012) and cytogenetics (Glick and Mayrose, 2014; Kolano et al., 2015; Van-Lume et al., 2017; Carta et al., 2018; Serbin et al., 2019), few papers have demonstrated their applicability in a geographic perspective (Carta and Peruzzi, 2016; Souza et al., 2019a). Thus, biogeographic analyses of ancestral area reconstruction (Matzke, 2012) using dated molecular phylogenies along with diversification rate analyses (see Menezes et al., 2017), may help to elucidate processes associated with karyotype diversification. This may be especially interesting in plant groups with ancient origin, intercontinental disjunct distribution and marked cytogenetic variability.

**FIGURE 1:**
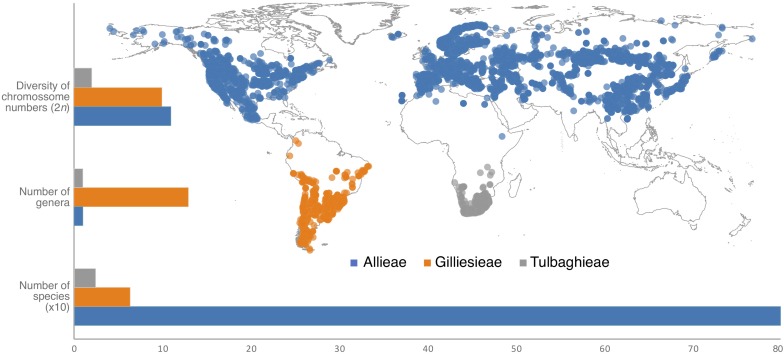
Geographic distribution and diversification patterns of the three tribes of the subfamily Allioideae (Amaryllidaceae). Geographic distribution was obtained from http://www.gbif.org.

The subfamily Allioideae of Amaryllidaceae (sensu Angiosperm Phylogeny Group [APG], 2016) is an excellent model to assess the links between karyotypes and environmental variables by presenting large chromosomes with remarkable cytogenetic variability (Vosa, 2000; Souza et al., 2016; Pellicer et al., 2017; Peruzzi et al., 2017; Sassone et al., 2018), associated with an intriguing discontinuous geographical distribution (Figure 1). Allioideae is phylogenetically composed of three tribes (Angiosperm Phylogeny Group [APG], 2016): Allieae, Gilliesieae, and Tulbaghieae, which are exclusively distributed in North Hemisphere, South America (Peru to Chile), and Southern Africa, respectively (Figure 1). The subfamily is composed of rhizomatous or bulbous geophytes, widely known for ornamental, medicinal or food use (e.g., chives, garlics, onions) (Pellicer et al., 2017). Allieae is represented by only *Allium* L. with ∼800 species (Friesen et al., 2006; Li et al., 2010; Hauenschild et al., 2017). South American tribe Gilliesieae with 80 species have traditionally been classified into two main groups, namely subtribes Gilliesiinae and Leucocoryninae, characterized by zygomorphic and actinomorphic flowers, respectively (Rudall et al., 2002; Chase et al., 2009; Escobar, 2012). Tulbaghieae is composed only of the monotypic *Tulbaghia* L. which comprises 26 Southern African species (Vosa, 2000; Chase et al., 2009; Stafford et al., 2016). A second genus, *Prototulbaghia*, has been proposed (Vosa, 2007), but it is deeply nested within the *Tulbaghia* clade (Stafford et al., 2016).

Most of the Allioideae genera can be distinguished by distinct and easily recognized karyological characteristics, making a cytotaxonomic approach particularly useful in this subfamily. They may have bimodal karyotypes (especially South American species), with large metacentric (M) and acrocentric (A) chromosomes, which are considered to be classical examples of karyotype evolution by Robertsonian translocations (RTs) or centric fusions/fissions (Jones, 1998). In general Allieae and Tulbaghieae are characterized by stability of the basic chromosome number, *x* = 8 and *x* = 6, respectively [except for few *Allium* species of the subgenera *Amerallium* Traub and *Melanocrommyum* (Webb & Berthel.) Rouy which present different chromosome numbers] (Peruzzi et al., 2017). Conversely, the South American Gilliesieae present the highest karyotype variability in Allioideae: it is represented by both different basic chromosome numbers (*x* = 4, 5, 6, 7, and 10) and chromosome numbers (2*n* = 8, 10, 12, 14, 15, 16, 18, 19, 20, 24, 26, and 32) resulted from intense Robertsonian translocation and polyploidy events (Escobar, 2012; Souza et al., 2016; Pellicer et al., 2017; Sassone et al., 2018). In addition, analyses of heterochromatic bands and distribution of 5S/35S rDNA sites by fluorescence *in situ* hybridization (FISH) have revealed extensive cytomolecular variability in the subfamily (Souza et al., 2016, 2019b). The different patterns of karyotype evolution and species-richness (i.e., high karyotype variation in low diversified lineages or stable karyotypes in high diversified groups) of Allioideae tribes (Figure 1) represent an interesting case study for the implications of intercontinental disjunction.

The current distribution of the three tribes of Allioideae indicates a few possible scenarios to explain its intercontinental disjunction based on the timing and place of their separation. A late Cretaceous split coupled with a Gondwanic origin would imply separation via continental drift, with an “Out-of-India” arrival of tribe Allieae in the Northern hemisphere (Briggs, 2003; Bossuyt et al., 2006; Datta-Roy and Karanth, 2009). A Laurasian origin following a mid-Eocene expansion through the Northern Hemisphere via the Boreotropical belt with late arrival in Africa and South America could also explain these disjunctions, as is the case with a number of angiosperms (Meng et al., 2014; Wei et al., 2015). Nevertheless, long distance dispersal has also often been appointed as a mechanism for intercontinental disjunction, especially in case of relatively young splits (Thiv et al., 2011; Crayn et al., 2014). In addition, if the two split events that formed the three Allioideae tribes are significantly apart, a combination of more than one of these processes could also be a likely scenario (Janssens et al., 2016; Yang et al., 2018). The reconstruction of the biogeographic history of Allioideae using a dated phylogeny may provide an adequate framework to discuss these different hypotheses and shed light on how the disjunction may have contributed to shape karyotype and species-richness patterns in the group.

We reconstructed the phylogeny of Amaryllidaceae, with a focus in Allioideae to assess its biogeographic and karyotype patterns. Additionally, we newly generate cytomolecular data (number and morphology of chromosomes and distribution of 5S and 35S rDNA sites) for Chilean genera of Gilliesieae: *Gethyum* Phil., *Miersia* Lindl., *Solaria* Phil., and *Speea* Loes. A molecular clock analysis was implemented to assess biogeographic hypotheses that might explain the disjunctions of the three Allioideae tribes. We specifically addressed three questions: (1) Why was karyotype evolution of Gilliesieae so variable (in terms of chromosome numbers and number/position of rDNA sites) compared to the other tribes of Allioideae? (2) Is Allioideae a Gondwanan or Laurasian group? (3) Can historical biogeographical events be related to the distinct karyotypic patterns of the Allioideae tribes?

## Materials and Methods

### Taxa Sampling

Karyotype and molecular data were collected from representatives of the subfamily Allioideae (Amaryllidaceae). Comparative cytogenetic analysis sampled 451 species from the three subfamilies of Amaryllidaceae: Amaryllidoideae (257 spp.) Agapanthoideae (4 spp.), and Allioideae [including the tribes Allieae (150 spp.), Gilliesieae (37 spp.), and Tulbaghieae (3 spp.)] (Supplementary Table S1). Newly generated cytomolecular data for seven species of Gilliesieae were included: *Gethyum atropurpureum* Phil., *Gethyum cuspidatum* (Harv. ex Baker) Muñoz-Schick, *Gilliesia graminea* Lindl., *Gilliesia montana* Poepp. & Endl., *Miersia chilensis* Lindl., *Solaria miersioides* Phil., and *Speea humilis* Loes. In addition, new genome size estimates were undertaken for genera *Ipheion* Raf., *Leucocoryne* Lindl., *Nothoscordum* Kunth, and *Zoellnerallium* Crosa [vouchers and collection locations presented in Souza et al. (2010, 2016)]. Collection locations, voucher numbers and karyotype data are presented in Table 1. The vouchers specimens were deposited at the herbarium CONC of the University of Concepción (Chile).

**TABLE 1:** Original data from cytogenetically analyzed species of the tribe Gilliesieae with voucher number, collect location, haploid chromosome number (*n*), Karyotypic formulae, Fundamental number (FN), and number of 5S and 35S rDNA sites number.

						**rDNA sites**	
**Species**	**Voucher**	**Collect location**	**2*n***	**Karyotypic formulae**	**FN**	**5S**	**35S**
*Speea humilis* Loes	CONC 30	Parque Nacional La Campana, Región de Valparaíso, Chile	6	4M + 1SM + 1A	11	2	2
*Gethyum cuspidatum* (Harv. ex Baker) Muñoz Schick	CONC 12	Parque Nacional Fray Jorge, Región de Coquimbo	7	4M + 1SM + 1A	11	2	6
*Gilliesia graminea* Lindl	CONC 18	Parque Nacional La Campana, Región de Valparaíso, Chile	7	2M + 2SM + 3A	11	2	6
*Gilliesia montana* Poepp & Endl	CONC 56	Reserva Los Ruiles de Empedrado, Región del Maule, Chile	7	2M + 2SM + 3A	11	2	6
*Miersia chilensis* Lindl	CONC 27	Parque Nacional La Campana, Región de Valparaíso, Chile	6	4M + 1SM + 1A	11	2	4
	CONC 95	Alhué, Región Metropolitana, Chile	10	1SM + 9A	11	6	26
*Solaria atropurpurea* (Phil.) Ravenna	CONC 4160	Quebrada Nido de Águila, Región Metropolitana, Chile	7	4M + 1SM + 1A	11	2	6
*Solaria miersioides* Phil	CONC 137	Los Álamos-Cipreses, Región del Maule, Chile	7	2M + 2SM + 3A	11	2	6
*Speea humilis* Loes	CONC 30	Parque Nacional La Campana, Región de Valparaíso, Chile	6	4M + 1SM + 1A	11	2	2

*M, metacentric; SM, Submetacentric; A, acrocentric.*

### Cytogenetic Analysis

Root tips from bulbs were pretreated with 0.05% colchicine for 24 h at 10°C, fixed in ethanol:acetic acid (3:1; v/v) for 2–24 h at room temperature and stored at −20°C. The fixed root samples were washed in distilled water and digested in 2% cellulase (Onozuka) and 20% pectinase (Sigma) at 37°C for 90 min. Subsequently, the apical meristem was squashed in 45% acetic acid under a coverslip. The material was fixed to the slide by deep freezing with liquid nitrogen, and the coverslip was removed perhaps with a razor blade. The FISH technique was used to located 5S and 35S rDNA sites following Souza et al. (2019b). The slides were mounted with DAPI (4 μg mL^–1^) diluted in Vectashield (Vector) 1:1 (v/v) and analyzed under an epifluorescence microscope (Leica DMLB). Images were recorded using a Cohu CCD camera and software Leica QFISH before editing with the software Adobe Photoshop CS3 v.10.0.

### Flow Cytometry

Absolute nuclear DNA contents were determined by flow cytometry according to Doležel et al. (2007). Fresh leaves from the specimens were collected to prepare the samples of 25–50 mg each. The material was chopped together with fresh leaf tissue of the internal standard (*Vicia faba* L. subsp. *faba* ‘Inovec’ 2C = 26.9 pg/2C DNA; Doležel et al., 1992) with a razor blade on a Petri dish (kept on ice) containing 1 mL of WPB isolation buffer (Loureiro et al., 2007). The solution was filtered through a 30 μm mesh filter and mixed with 50 μg/mL of propidium iodide (1 mg/mL).

Flow cytometry measurements were taken using a Partec Cyflow Space (Müster, Germany) equipped with a 488 nm laser canon. The relative fluorescence histograms were analyzed on FloMax program version 2.3. The coefficient of variation of obtained peaks was assessed at half of the peak height (H.P.C.V.), discarding peaks with a H.P.C.V. > 5%. The genome size (ρg) of the samples were calculated using the following equation: “sample DNA = (sample G1/standard G1) × standard DNA,” where sample G1 is the peak position (G1) of the sample; standard G1 is the peak position (G1) of the standard, and standard DNA is the nuclear DNA (ρg) of the standard used in each measure. Three independent DNA estimations were performed on different days for each sample. Measurements were exhausted with at least 1,500 events per fluorescence peak.

### Cytogenetic Data Survey

We surveyed additional data for chromosome number, genome size and 35S and 5S rDNA sites for species of Allioideae, Agapanthoideae and Amaryllidoideae (Plant rDNA database^1^). The chromosome number of 448 species was obtained from the Chromosome Count Database v. 1.46^2^ (Rice et al., 2015) (Table 1 and Supplementary Table S1). The ratio of chromosome arms (AR = the long arm length/short arm length) was used to classify the chromosomes as metacentric (M; AR = 1–1.4), submetacentric (SM; AR = 1.5–2.9), or acrocentric (A; AR > 3.0), following Guerra (1986).

**FIGURE 2:**
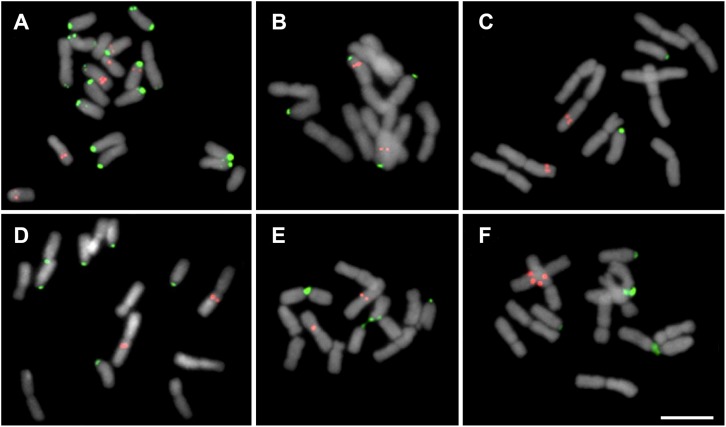
Mitotic cells showing the distribution of 5S (red) and 35S (green) rDNA sites in Chilean Gilliesieae species. **(A,B)** Individuals of *Miersia chilensis* com 2*n* = 20 (2SM + 18A) **(A)** and 2*n* = 12 (8M + 2SM + 2A) **(B)**. **(C)** *Speea humilis* 2*n* = 12 (8M + 2SM + 2A). **(D–F)** Species with 2*n* = 14 (4M + 4SM + 6A): *Gilliesia graminea* **(D)**, *Gethyum atropurpureum* **(E)**, and *Solaria miersioides* **(F)**. Scale bar in **(F)** = 10 μm.

For genome size, we compiled data for 189 species from the Kew Gardens C-Value Database v. 6.0^3^ (Bennett and Leitch, 2012) along with 62 records from literature that were not in the database and 13 new estimates (Table 1 and Supplementary Table S1). We plotted genome sizes and chromosome numbers for Allioideae in dispersion plots using the package *stats* of the software R (R Core Team, 2013) (see Figure 2).

We also surveyed number and position of 35S and 5S rDNA sites for 55 and 59 species, respectively (Supplementary Table S1). Metaphasis pictures and/or original idiograms with scale information (when available) were used to construct a simplified idiogram based on Lima-de-Faria (1976) containing only the site-bearing chromosomes of the species surveyed. All chromosomes measurements were made using Adobe Photoshop CS3 and the idiogram was drawn on CorelDraw X7.

### Phylogenetic Analyses

To provide a robust phylogenetic framework for the subsequent analyses, we reconstructed a phylogenetic tree sampling taxa from the three subfamilies of Amaryllidaceae, of which 190 were Allioideae species and 261 species were from Agapanthoideae (4 spp.) and Amaryllidoideae (257 spp.). *Aloe vera* (L.) Burm.f. (Xanthorrhoeaceae) was used as outgroup (Supplementary Table S1). We used available data for one nuclear (ITS) and three plastids (*mat*K, *ndh*F, and *rbc*L) loci from GenBank (see accession numbers in Table 1 and Supplementary Table S1), with each species having at least one locus sampled. Missing data were coded as gaps and accounted for 20.5% of the matrix.

An aligned matrix including data from the four markers was obtained using MUSCLE as a plugin implemented in Geneious v.7.1.9 (Kearse et al., 2012). We used jModelTest v.2.1.6 to assess the best-fit model of DNA substitution for each marker (Darriba et al., 2012) through the Akaike Information Criterion (AIC; Akaike, 1974). The selected models were SYM + I + G for ITS, HKY + G for *rbc*L, and GTR + G for *mat*K and *ndh*F.

Phylogenetic relationships were inferred using Bayesian Inference (BI) implemented in MrBayes v.3.2.6 (Ronquist et al., 2012). The analyses were performed on the combined data set, specifying the substitution model for each marker. Four independent runs with four Markov Chain Monte Carlo (MCMC) runs were conducted, sampling every 1,000 generations for 100,000,000 generations. Each run was evaluated in TRACER v.1.6 (Rambaut et al., 2018) to determine that the estimated sample size (ESS) for each relevant parameter was higher than 200 and a burn-in of 25% was applied. We then obtained the consensus phylogeny and clade posterior probabilities with the “sumt” command (contype = allcompat). The tree was visualized and edited in FigTree v.1.4.2 (Rambaut, 2014). All BI and jModelTest analyses were performed on the CIPRES Science Gateway (Miller et al., 2015).

### Divergence Time Estimates

A molecular clock analysis was performed to explore the karyotype and biogeographic evolution in Allioideae. Divergence times were estimated on BEAST v.1.8.3 (Drummond and Rambaut, 2007) through CIPRES Science Gateway fixing the tree topology from the BI. Uncorrelated relaxed lognormal clock (Drummond and Rambaut, 2007) and Birth-Death speciation model (Gernhard, 2008) were applied. Two independent runs of 100,000,000 generations were performed, sampling every 10,000 generations. After removing 25% of samples as burn-in, the independent runs were combined and a maximum clade credibility (MCC) tree was constructed using TreeAnnotator v.1.8.2 (Rambaut and Drummond, 2013). In order to verify the effective sampling of all parameters and assess convergence of independent chains, we examined their posterior distributions in TRACER. The MCMC sampling was considered sufficient at effective sampling sizes (ESS) equal to or higher than 200.

The phylogeny was dated using one macrofossil of Amaryllidaceae and one secondary calibration from published dated phylogenies. The first fossil calibration was based on a fossil leaf attributed to Amaryllidaceae from the Cerrejón Formation, Colombia, estimated at 58 Mya (Wing et al., 2009). This fossil calibration was used to set the minimum age for Amaryllidaceae diversification. The second calibration point was based on a *rbc*L phylogeny of 554 angiosperms genera, which estimated the Amaryllidaceae crown group to be 59.6 Mya (BEAST analysis; Hermant et al., 2012). From the age variability suggested for the secondary calibration (33–59.6 Mya) we followed Hermant et al. (2012) due to their broadly sampled analysis with an age estimate more consistent with the fossil age.

### Ancestral Range and Biogeographic Events Estimation

To investigate the historic biogeography of Amaryllidaceae, we employed a model-based likelihood approach implemented in the R package BioGeoBEARS (Matzke, 2013, 2014). The sampled species from the MCC tree yielded by BEAST were coded as present or absent in nine discrete areas around the globe: Africa, Andean region, Asia (India, west and east Asia), Europe + Siberia, Mediterranean region, Mesoamerica (Mexico + Central America), North America (minus Mexico), Oceania, and South America (minus Andean region). The regions were coded based on a search on the Global Biodiversity Information Facility website^4^. For ancestral range estimation, we used the MCC tree to test likelihood implementations of three different biogeographic models in BioGeoBEARS. In order to better reflect geological events through time, we stratified the tree in five time periods based on important events: (i) 70 to 55 Mya: period when the Indian subcontinent is completely separated of the African continent and migrates to the Laurasia, which is still in early separation; (ii) 55 to 40 Mya: Both Gondwana and Laurasia completed separation in two smaller land masses, the Indian subcontinent is completely connected with Asia and the Himalayans and Andean uplifts are in motion; (iii) 40 to 18 Mya: The Andes and the Himalayans continue to grow and change the South American and Asian landscape, Africa and Europe are closer each other; (iv) 18 to 3 Mya: Africa and Europe are connected by the Gibraltar strait and the Mediterranean region is fully established with its own characteristic climate; (v) 3 Mya to the present: The Isthmus of Panama connects the Americas. Both the dispersal probability and connection between areas in each of these periods was adjusted accordingly. Based on this, we informed different dispersion probabilities between areas for each time slice, following Buerki et al. (2011): low dispersal = 0.01; medium dispersal = 0.5; high dispersal (including areas adjacent or very close) = 1.0. We compared the results of models with and without the parameter j using likelihood ratio tests and the model weights were calculated under the AIC. To illustrate the geological state of the earth in different time periods, we generated paleomaps using the web tool available at http://www.odsn.de/odsn/services/paleomap/paleomap.html and edited the maps with the software CorelDraw X7.

### Ancestral Character Reconstruction

Literature and newly generated chromosome number data were used to reconstruct the chromosome number evolution of the family along the MCC tree. To assess the events and processes (for instance RTs) that may have fostered the karyotype diversity across Amaryllidaceae phylogeny, we employed a statistical framework. We applied ChromEvol to test whether karyotypes evolved by polyploidy or dysploidy (Glick and Mayrose, 2014). The best fitting model was assessed using the AIC (Glick and Mayrose, 2014). The best fitted model was used to reconstruct the chromosome number along the MCC tree of Amaryllidaceae and two simplified trees with key events and ancestral numbers were drawn on CorelDraw X7.

In order to investigate the mode of evolution for chromosome number in each Allioideae tribe, we used the function *fitContiuous* implemented in the R package *geiger* (Harmon et al., 2008). Individual trees for each lineage (Gilliesieae, Tulbaghieae, *Allium* I, II, and III) were obtained by pruning the MCC tree with the function *drop.tip* implemented in the R package *phytools* (Revell, 2012). We fitted nine different likelihood models of continuous character evolution for each lineage and compared the results using AIC: (i) Brownian motion model - BM (Felsenstein, 1973); (ii) Ornstein-Uhlenbeck model - OU (Butler and King, 2004); (iii) Early-burst model - EB (Harmon et al., 2010); (iv) trend model; (v) lambda model (Pagel, 1999); (vi) kappa model (Pagel, 1999); (vii) delta model; (viii) drift model; (ix) white model (for details^5^).

We also reconstructed the 35S rDNA sites number as a continuous character along a simplified phylogeny (Figure 1B). Species without information for this site were pruned off from the tree with the function *drop.tip* implemented in the package *phytools* on R and the ancestral reconstruction was made with the function *cont.map*, also in *phytools*.

### Diversification Rate Analysis

Shifts in diversification rates were calculated using speciation/extinction model type analysis in BAMM (Rabosky et al., 2014). To work with incomplete phylogenetic datasets in BAMM, it is necessary to input the percentage of sampled species for each major clade. This percentage was estimated according to the total number of accepted names reported for each subtribe (WFO, 2019). For this, tribe Allieae was divided in the three different lineages proposed by Friesen et al. (2006). Percentage of sampled species per tribe as informed on BAMMtools is presented in the Supplementary Table S2. Priors for the BAMM control file were generated using the dated phylogenetic tree input into the function *set BAMM priors* in the package BAMMtools v. 2.5.0 implemented in R. The control file was set for 10,000,000 generations and the analysis was run twice as recommended, returning similar results. Resulting MCMC log likelihoods were tested against generation number using the *CODA* package (Plummer et al., 2006) implemented in R. All remaining outputs contained in the event data file were analyzed using BAMMtools. BAMMtools was then used to produce a figure showing the best rate shift configuration as well as graphics of diversification through time for Gilliesieae + Tulbaghieae and each of the three evolutionary lineages of Allieae.

## Results

### Cytomolecular Characterization of Chilean Gilliesieae

The analysis of the seven Chilean Gilliesieae species revealed large (5.5–14.9 μm) chromosomes which were metacentric (M) or submetacentric (SM) or acrocentric (A). Three distinct chromosome complements with the same number of chromosome arms or fundamental number NF = 11 were observed: 2*n* = 12 (8M + 2SM + 2A), 2*n* = 14 (4M + 4SM + 6A), and 2*n* = 20 (2SM + 18A). FISH with the 35S rDNA probe revealed signals on the short arm of the acrocentric chromosomes (Figure 1).

Two different cytotypes were observed in *Miersia chilensis*. The individual collected in the municipality of Santiago (Chile) presented 2*n* = 20 (2SM + 18A) with a large 5S rDNA site in the interstitial region of one acrocentric pair and a smaller extra 5S rDNA site in the proximal region of two additional acrocentric pairs (Figure 1A). However, the individual collected in the municipality of Valparaiso (Chile) presented 2*n* = 12 (8M + 2SM + 2A) with 5S rDNA sites in the interstitial region of the long arm of the largest metacentric pair (Figure 1B), a karyotype similar to that observed in *Speea humilis* (Figure 1C). *Gethyum atropurpureum*, *Gethyum cuspidatum*, *Gilliesia graminea*, *Gilliesia montana*, and *Solaria miersioides* presented 2*n* = 14 (4M + 4SM + 6A) and very similar karyotypes with 5S rDNA sites near the centromere of a pair of metacentric chromosomes (Figures 1D–F).

### Cytogenetic Variability of Amaryllidaceae

**FIGURE 3:**
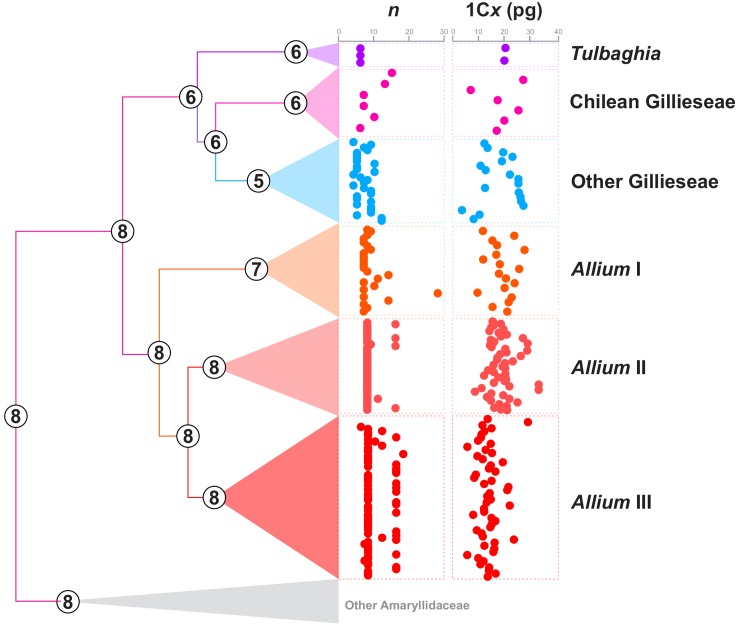
Ancestral chromosome number reconstruction inferred with ChromEvol of Amaryllidaceae species. Numbers at nodes represent the most probable ancestral chromosome number (*n*). Dispersion plots at the right of the phylogeny represent the chromosome numbers and genome sizes (1C_x_ values) of the different Allioideae groups.

The haploid chromosome number (*n*) varied in Amaryllidaceae from *n* = 4 on *Nothoscordum pulchellum*, *Tristagma bivalve* and *Tristagma graminifolium*, to *n* = 68 in *Eucharis amazonica* (Supplementary Table S1 and Figure 3). Gilliesieae was represented by only 36 records and ten different chromosome numbers (2*n*). On the other hand, 148 records were obtained to Allieae (11 different 2*n*, most of which polyploid series). For Tulbaghieae only three different 2*n* were recovered, with stability 2*n* = 12.

For genome size we accomplished 13 new estimates for species of Gilliesieae (Supplementary Table S1). In the subtribe Gilliesiinae, both *Speea humilis* and *Gilliesia gramina* presented large genome sizes (2C = 56.65 and 36.04 pg, respectively) as observed in most of Allioideae. The estimates for *Ipheion* presented two of the smallest genome sizes in the subfamily [*I. recurvifolium* (2C = 18.1 pg) and *I. uniflorum* (2C = 18.06 pg)]. The estimates for genera *Leucocoryne* and *Nothoscordum* showed large genomes as frequently observed for these two genera (Supplementary Table S1). For the subsequent analysis, the monoploid genome size value (1C*x*) was obtained by dividing the 2C value by the ploidy level, also informed on the 2C value database. The 1C*x* value varied from 1C*x* = 4.52 in *Ipheion uniflorum* to 1C*x* = 65.45 in *Sprekelia formosissima*. In this case, the genome size varied 6.3-fold among 25 records of Gilliesieae, whereas in Allieae the variation was of 4.5-fold among 126 records (Supplementary Table S1 and Figure 3).

**FIGURE 4:**
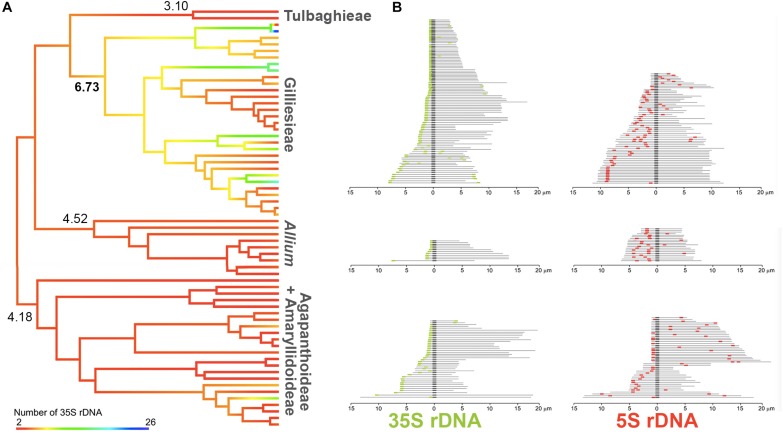
Evolution of rDNA sites across Amaryllidaceae phylogeny. **(A)** Ancestral number of 35S rDNA sites reconstruction on a pruned phylogeny of Amaryllidaceae. Hot branch colors indicate higher number of sites while cold colors represent lower number of sites. **(B)** Schematic idiograms showing the position of 5S (red) and 35S (green) rDNA sites on the chromosomes of species of the subfamily Allioideae. Darker regions represent the centromere.

According to our Chromevol analysis, all these three events were almost equally important to karyotype evolution (*f* = 93 for chromosome gains, 83.1 for chromosome losses, and 86.5 for duplications). The ancestral haploid number for Amaryllidaceae was *n* = 8 (pp = 0.50), with the loss of one chromosome originating an ancestral haploid number *n* = 7 (pp = 0.45) for Allioideae (Figure 4). The Allieae was characterized by gain of one chromosome (*n* = 8, pp = 0.61), while Tulbaghieae and Gilliesieae derived from a shared ancestor with *n* = 6 (pp = 0.94). The MRCA of Tulbaghieae retained *n* = 6 (pp = 0.99). The MRCA of the Chilean Gilliesieae also retained *n* = 6, though with a high variability despite its fewer number of extant taxa (Figure 1). The other Gilliesieae species were marked by the loss of one chromosome in its MRCA (*n* = 5, pp = 0.56) and also a high number of karyotype events in more recent splits. Among the lineages of *Allium*, only the first presented a change on the haploid chromosome number of the ancestral node, showing *n* = 7 (pp = 0.99), with this number being conserved on most of its taxa. Both the second and third taxa retained *n* = 8 (pp = 0.99), with the former being extremely conserved, while the latter presented high incidence of polyploidy. Our analysis of continuous character evolution revealed that chromosome number evolution likely followed distinct modes in each lineage (Supplementary Table S3). The best-fitted model for tribe Gilliesieae was a model based on Pagel’s ‘Lambda’ (Pagel, 1999) which assumes that trait variation is associated with phylogenetic relatedness. For Tribe Tulbaghieae, the best-fitted model was based on the time-dependent parameter ‘Delta’ (Pagel, 1999). The delta model fits the relative contributions of early versus late evolution in the tree to the covariance of species trait values. On Tribe Allieae, chromosome evolution of *Allium* I and II was better explained by the ‘White’ model, which implies that trait variation has no phylogenetic meaning. Meanwhile, the best-fitted model for *Allium* III was the ‘Ornstein-Uhlenbeck’ model (Butler and King, 2004), which implies that trait variation fits a random walk toward an “evolutionary optimum” state in different lineages.

For the rDNA survey, 60 records (including the seven original FISH results reported here) were observed for 35S rDNA ranging from 2 to 26 sites, while 64 records were observed for 5S sites number, varying from 2 to 16 (Supplementary Table S1). The 5S rDNA sites were scattered along different regions of the chromosomes. Duplicate sites in the interstitial region of the same chromosomal arm were consistently observed for the three subfamilies of Amaryllidaceae (Figure 4). In turn, the 35S rDNA sites showed a tendency to appear on the short arm of acrocentric chromosomes (Figure 4).

To assess the evolution of 35S rDNA, we treated it as a continuous character (Figure 4). Agapanthoideae, Amaryllidoideae, Allieae, and Tulbaghieae presented little variation on number of sites, with similar inferred ancestral numbers (four to five sites). In contrast, Gilliesieae had great variability on 35S site number and a noticeable increase was observed on the inferred ancestral of the tribe, presenting six to seven sites (Figure 4).

### Phylogenetic Relationships and Historical Biogeography of Amaryllidaceae

**FIGURE 5:**
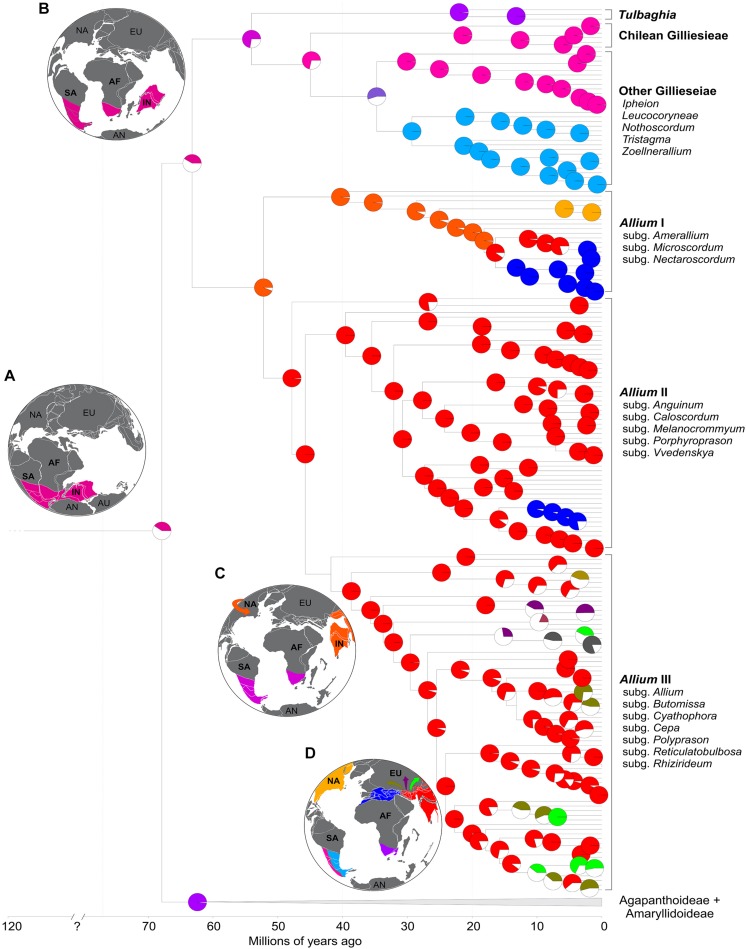
Ancestral range reconstruction for Amaryllidaceae (under the DEC + J model) focusing on subfamily Allioideae. Pie charts at the nodes represent the probability of ancestral range. Colored slices represent the most probable ancestral range while white slices represent other possible ranges. Panels **(A–D)** are paleomaps representing the geological state of the Earth at 120, 65, 40, and 20 Mya, respectively.

The most recent common ancestors (MRCAs) of Amaryllidaceae, the three subfamilies and the three Allioideae tribes presented high support values (pp > 0.95) while most internal nodes presented moderate to low support. The DEC model with the addition of the free parameter j presented the most likely biogeographic scenario for the family (LnL = −723.55). According to our data, the crown node of Amaryllidaceae appeared 67.9 Mya (77.2–58.5 Mya: 95% HPD), with a probable Gondwanan stem age and distribution in parts of South America, Africa, and India (Figure 5A). From there, the three main subfamilies followed different evolutionary paths. The MRCA of Agapanthoideae and Amaryllidoideae diversified from Africa approximately 62.7 Mya (68.4–55.8 Mya: 95% HPD), with the former remaining in Africa, while the latter colonized regions of Europe, Asia and South America. Allioideae splitted shortly after separating from the other subfamilies 63.2 Mya (67.5–53.7 Mya: 95% HPD) (Figure 5B). One of the lineages became Allieae 52.2 Mya (58.1–44.4 Mya: 95% HPD), rapidly colonizing parts of Asia and North America after arriving presumably via the Indian Subcontinent (Figure 5C). From there, Allieae colonized most regions of the Northern hemisphere (Figure 5D). The other lineage splitted 54.1 Mya (65.1–37.11 Mya: 95% HPD) into Tulbaghieae and Gilliesieae. Tulbaghieae did not expand from Africa while Gilliesieae diversified in South America, splitting further into the more widespread Gilliesiinae and the Chilean Andean Leucocorynae approximately 45 Mya (61.2–32.2 Mya: 95% HPD).

### Diversification Rate Shifts

**FIGURE 6:**
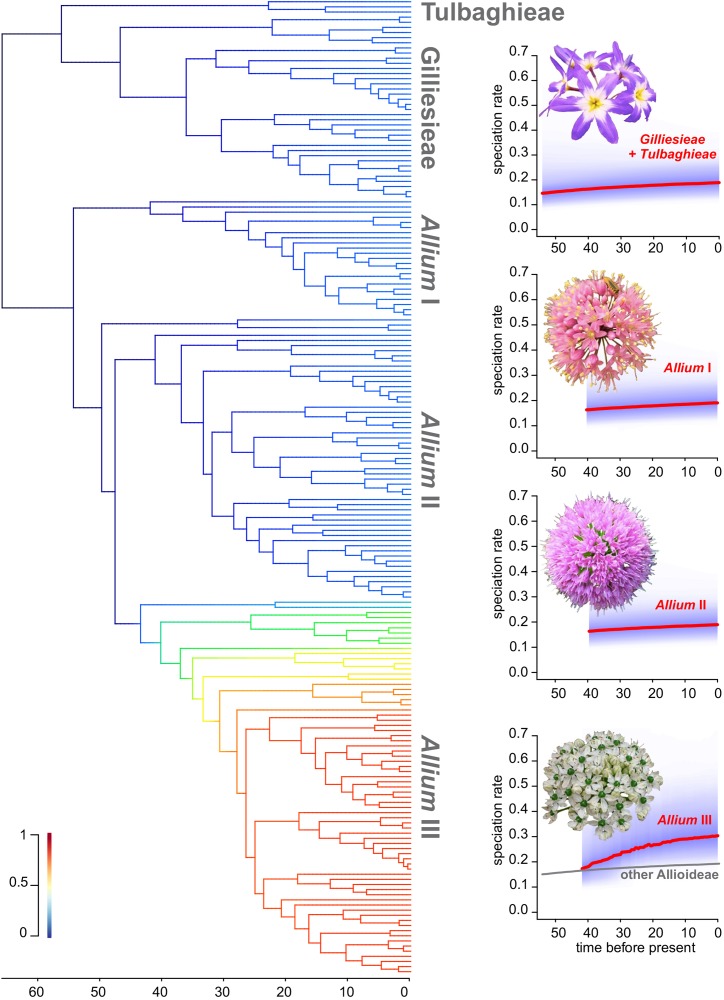
Rate of diversification through time on the subfamily Allioideae. On the left, the dated phylogeny of the subfamily, with colored branches indicating the rate of diversification. Colder colors represent low rates, while hot colors represent high rates. On the right, plots of diversification rate through time for each Allioideae lineage. Strong red lines represent the consensus correlation, while purple shade represents the uncertainty of the analysis.

The 95% credible set of rate shift configurations yielded by BAMM showed seven possible shift configurations, always with one shift on a different early node of the third evolutionary lineage of *Allium*. For better visualization, a mean phylorate was obtained, showing a continuous increase on diversifications rate on this lineage (Figure 6). To further explore the difference in diversification rate, four different density plots of speciation through time were obtained. It was evidenced a steady increase on diversification in the third evolutionary lineage of *Allium* (III) since its origin circa 42 Mya. Meanwhile, Gilliesieae + Tulbaghieae and *Allium* I + II presented a more constant speciation rate through time (Figure 6).

## Discussion

### Centric Fissions Are the Key Mechanism to Explain the High Karyotype Diversity in Gilliesieae

The maintenance of the number of chromosome arms accompanied by chromosomal number (2*n*) changes in Chilean Gilliesieae species analyzed here clearly indicates a karyotype evolution by Robertsonian translocations Escobar, 2012). This type of chromosomal rearrangement was also observed other genera of Gilliesieae (Crosa, 1972; Jones, 1998; Souza et al., 2010, 2012, 2015, 2016, 2019b), however, it is rarely reported in species of Allieae or Tulbaghieae (Vosa, 2000; Peruzzi et al., 2017). Interestingly, our data also suggests that chromosome number evolution is more associated with phylogenetic relatedness in Gilliesieae than in the other tribes (Supplementary Table S1), which reinforces the impact of chromosome number change for the evolution of this tribe. Morphoanatomic and phylogenetic analyzes suggest that *Miersia* and *Speea* are the first diverging lineages of Gilliesieae (Rudall et al., 2002; Escobar, 2012; Pellicer et al., 2017) corroborating our phylogenetic hypothesis (see Figure 5). Thus, the 2*n* = 12 karyotype observed in these genera may represent a plesiomorphic condition for Gilliesieae, shared with *Thulbaghia* (Vosa, 2000).

Across the Allioideae subfamily karyotypes are predominantly formed by metacentric chromosomes as reported for *Allium* (Peruzzi et al., 2017), *Leucocoryne* (Souza et al., 2015), *Nothoscordum* (Souza et al., 2019b), and *Tristagma* (Crosa, 1981). Only *Ipheion* (three species; Souza et al., 2010) and *Zoellnerallium* (two species; Souza et al., 2016) have acrocentric-rich karyotypes, suggesting that this is a derived condition. In this sense, *Miersia chilensis* samples with 2*n* = 20 and 2*n* = 21 (Cave and Bradley, 1943) may represent recent events of multiple centric fissions. Pellicer et al. (2017) analyzing Gilliesieae species reported the impact of multiple Robertsonian translocations on the reductions in the overall genome size. However, it is unclear how often such centric fissions lead to genomic expansions or contractions (Pellicer et al., 2017). This explains the high variability in genome sizes observed here in South American Gilliesieae.

Interestingly, the Robertsonian translocations seem do not affect the number of 5S rDNA sites, with one site per monoploid assembly being observed in most species of the tribe (Souza et al., 2012, 2016). Conversely, the number and position of 35S rDNA sites were directly affected by Robertsonian translocations, with the formation of new 35S rDNA sites in the short arms of the acrocentric (see Hall and Parker, 1995). This trend led to an increase in the number of rDNA sites in Gilliesieae when compared to other tribes of Allioideae (see Figure 3). This correlation between centric fissions and increase in the number of 35S rDNA sites in the acrocentric short arms has also been reported in other plant genera (Tagashira and Kondo, 2001), mollusks (Pascoe et al., 1996) and insects (Nguyen et al., 2010), suggesting that this may be an inherent feature of the mechanism of centric fission in eukaryotes.

### Historical Biogeography of Allioideae Support the Role of Indian Plate Carrying Allieae to Northern Hemisphere

Our data support that the current intercontinental disjunction of the Allioideae tribes (see Figure 1) may have been the result of vicariance after Gondwanan breakup (Givnish and Renner, 2004; Bartish et al., 2011). The fact that Allioideae are geophyte plants and usually without specialization for long-range dispersion and the old age of the phylogenetic splits that formed the three tribes reinforces the hypothesis of vicariance. This is corroborated by BioGeoBEARS analysis that revealed a predominance of vicariant events compared to few long-range dispersal events (concentrated in the *Allium* III clade). Our estimate Amaryllidaceae crown age 67.9 Mya (77.2–58.5 Mya: 95% HPD) suggests that the family may be much older than the secondary calibration previous estimates (∼33 Mya: Bremer, 2000; ∼50 Mya: Chen et al., 2013). This hypothesis of old-aged Amaryllidaceae is corroborated by their only macrofossil collected in the Cerrejón Formation, Colombia, estimated at 58 Mya (Wing et al., 2009) and by an extensive molecular clock analysis covering 800 monocots, which concluded that Allioideae has crown age 87 Mya and stem age 91 Mya (Janssen and Bremer, 2004). Specifically in *Allium*, the divergence times shown here (∼52 Mya) are also much older than reported in the literature (11 Mya to 34.25 Mya: Li et al., 2010; Chen et al., 2013; Hauenschild et al., 2017) based on secondary calibrations.

Our molecular clock and ancestral area reconstruction analysis, as well as the presence of the only Amaryllidaceae fossil in South America (Wing et al., 2009), suggest a Gondwanic origin of Allioideae. All Gondwanan breakup models suggest that the physical separation between Africa, South America, and India occurred sometime during the end of the Early Cretaceous or earliest Late Cretaceous (∼110–70 Mya). Our median crown age estimate for Amaryllidaceae is 67.89 Mya, with a variance from 77.26–58.51 Mya (95% HPD). Because the upper age estimate is situated within the 110–70 Mya range assumed for the Gondwanan continents (Figure 5), we cannot reject the hypothesis that the split ‘Gilliesieae + Tulbaghieae → Allieae’ and ‘Gilliesieae → Tulbaghieae’ resulted from the rifting of Africa, South America and India tectonic plates.

After the Gondwana breakup the Indian plate supposedly underwent a period of isolation [30–40 Mya] moving north, before colliding with the Eurasian plate around 40–50 Mya (Datta-Roy and Karanth, 2009). Consequently, the “Biotic ferry model” was proposed, according to which the rafting Indian plate carried ancient Gondwanan forms to Asia (Briggs, 2003; Bossuyt et al., 2006). After India collided with the Asian continent in the Early Tertiary, a few surviving Gondwanan elements dispersed out of India into South and Southeast Asia, which at the time lay in the same latitudinal and climatic zone (Morley, 2000). The out-of-India hypothesis adjusts to the arrival of *Allium* in the northern hemisphere in view of the age of the group (∼52 Mya compatible with the collision of the Indian and Eurasian plate) and by the center of origin of *Allium* in eastern Asia identified here. Friesen et al. (2006) identify also three main clades in *Allium*, with the first diverging lineages *Nectaroscordum* and *Microscodum* with center of origin Mediterranean and eastern Asia, respectively. Similarly, Li et al. (2010) proposed an *Allium* origin in eastern Asia (Northwest China), a geographic region of high species diversity for the genus, which corroborates our results. Interestingly, the genus *Allium* is not currently distributed in peninsular India (except Himalaya), a pattern similar to that seen in other ‘out of Indian’ groups (Datta-Roy and Karanth, 2009). It is argued that the extinction of these lines in India due to aridification and drastic climate change that occurred in India upon collision with Eurasia (Karanth, 2003).

### Evolutionary History of Each Allioideae Lineage Impacts in Tribe-Specific Trends of Diversification

Our data suggests that the evolutionary history of each Allioideae lineage impacts in tribe-specific trends of diversification. The long time of origin, stable diversification rates, and relatively low number of species in Gilliesieae (80 species) and Tulbaghieae (26 species) may suggest that these are relictual lineages. In this sense, the scenario of multiple geomorphological changes in South America, mainly caused by the Andean uplift (Antonelli et al., 2009; Pennington et al., 2010), may have been responsible for phylogenetic, karyotype and morphological differentiation in Gilliesieae species (Rudall et al., 2002; Pellicer et al., 2017; Sassone et al., 2018). The origin of the Andes is related here to the isolation of the Chilean clade with strong differentiation in floral morphology (Rudall et al., 2002; Escobar, 2012) and karyotypes. On the other hand, southern Africa experienced a scenario of more recent tectonic stability (Hälbich, 1992), which might be related to low morphological and karyotype diversification of *Tulbaghia*.

Similarly, diversification patterns in *Allium* seem to reflect coherence between biogeography and karyotype evolution. Accordingly, our continuous character evolution analysis suggests very different modes of karyotype evolution for the three main tribes of Allioideae (Supplementary Table S3). The colonization of northern hemisphere should have favored a higher diversification rate in *Allium* (Figure 6) associated with increased polyploidy and territorial expansion to Europe and North America. This geographic expanding trend is especially pronounced in the most recent *Allium* clade (III), which in light of the mid-Eocene date of the crown node could present an example of range expansion through the Boreotropical belt. Although the relationship between polyploidy and geographic expansion, especially colonization of new environments, is widely reported (Souza et al., 2012), the impact of genomic duplication on the rate of diversification is controversial, as some analyses have shown that the increase of polyploidy does not led necessarily to increased diversification rate (Sader et al., 2019). This suggests that colonization of the northern hemisphere by *Allium* was a complex and long-time process, accompanied by intense morphological diversification, which resulted in a species-rich genus with a complex taxonomic delimitation (Friesen et al., 2006).

## Conclusion

Historical biogeographic analysis including ancestral area reconstruction, dated molecular phylogeny and diversification rate analysis, were used here to unravel the karyotypic evolution of Allioideae (Amaryllidaceae). Our data support that the current intercontinental disjunction between the three tribes of Allioideae may have been the result of vicariance due to Gondwanan breakup. The results point to the possibility that the Indian plate carried Allieae to northern hemisphere (‘out-of-India’ hypothesis). From there, the genus *Allium* diversified through polyploidy and geographic expansion in North Hemisphere. Interestingly, karyotype stability in Allieae (predominantly 2*n* = 16) and Tulbaghieae (predominantly 2*n* = 12) are probably results of two distinct processes: recent colonization in North Hemisphere and relictual distribution in south Africa, respectively. On the other hand, the South American tribe Gilliesieae (*x* = 6) varied widely in genome size, chromosome number and rDNA sites distribution mainly related to Robertsonian translocations.

## Data Availability Statement

All datasets generated and analyzed for this study are included and cited in the article/Supplementary Material.

## Author Contributions

LC, HJ, and GS designed the study. GS and LC carried out FISH and flow cytometry experiments, respectively. JC-S and IE reviewed phylogenetic analyses. IE collected and identified Chilean Gilliesieae species. GS, LC, JC-S, and RC performed data analysis and wrote the manuscript. All authors read and approved the manuscript.

## Conflict of Interest

The authors declare that the research was conducted in the absence of any commercial or financial relationships that could be construed as a potential conflict of interest.

## Footnotes

1 http://www.plantrdnadatabase.com

2 http://ccdb.tau.ac.il/

3 https://cvalues.science.kew.org/

4 gbif.org

5 http://www.webpages.uidaho.edu/l̃ukeh/software.html

## Supplementary Material

FIGURE S1 Ancestral haploid chromosome number reconstruction in Amaryllidaceae. Pies charts at the nodes represent the probabilities of the inferred numbers with the most probable number shown inside the pie. Color coding is explained at the left side captions. Numbers above branches represent the posterior probability of different chromosome number events as explained in the botton left caption.

TABLE S1 List of Amaryllidaceae species, haploid chromosome numbers (*n*), genome size of the diploid chromosome complement (2C) in picograms (pg), number of 35S and 5S rDNA sites, references for both 2C and rDNA sites number and GenBank accession numbers for the four regions used in this work. * = 2C values obtained from the Royal Botanic Gardens Kew C-value database (https://cvalues.science.kew.org/, Bennett and Leitch, 2012).

TABLE S2 Number of species sampled for BAMM analysis and percentage relative to the total number of species reported for tribes Tulbaghieae, Gilliesieae (divided in subtribes Leucocoryninae and Gilliesiinae) and Allieae (divided in the three *Allium* evolutionary lineages).

TABLE S3 Results from the evolutionary model analysis for chromosome number and genome size with Log-Likelihood (Log-Lk) and AIC scores for each of the nine models. The analysis were repeated seven times for different combinations of taxa. Most likely models are highlighted in bold.
