# Supplementary material for: Divide to Conquer: Evolutionary History of Allioideae Tribes (Amaryllidaceae) Is Linked to Distinct Trends of Karyotype Evolution
Source: Front Plant Sci. 2020 Apr 7;11:320. doi: 10.3389/fpls.2020.00320 (PMC7155398; doi:10.3389/fpls.2020.00320)
Supplement: FIGURE S1 — Ancestral haploid chromosome number reconstruction in Amaryllidaceae. Pies charts at the nodes represent the probabilities of the inferred numbers with the most probable number shown inside the pie. Color coding is explained at the left side captions. Numbers above branches represent the posterior probability of different chromosome number events as explained in the botton left caption. [file Data_Sheet_1.PDF]

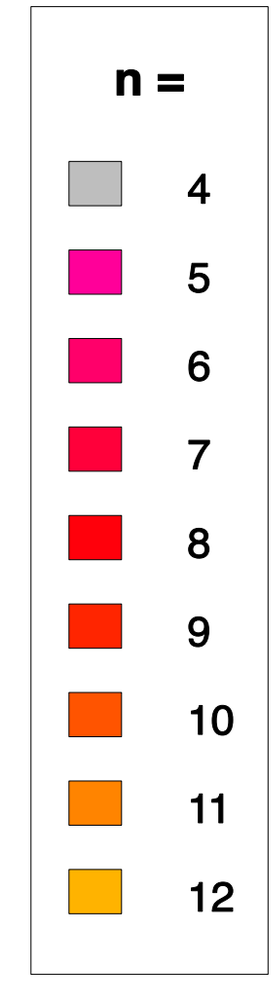

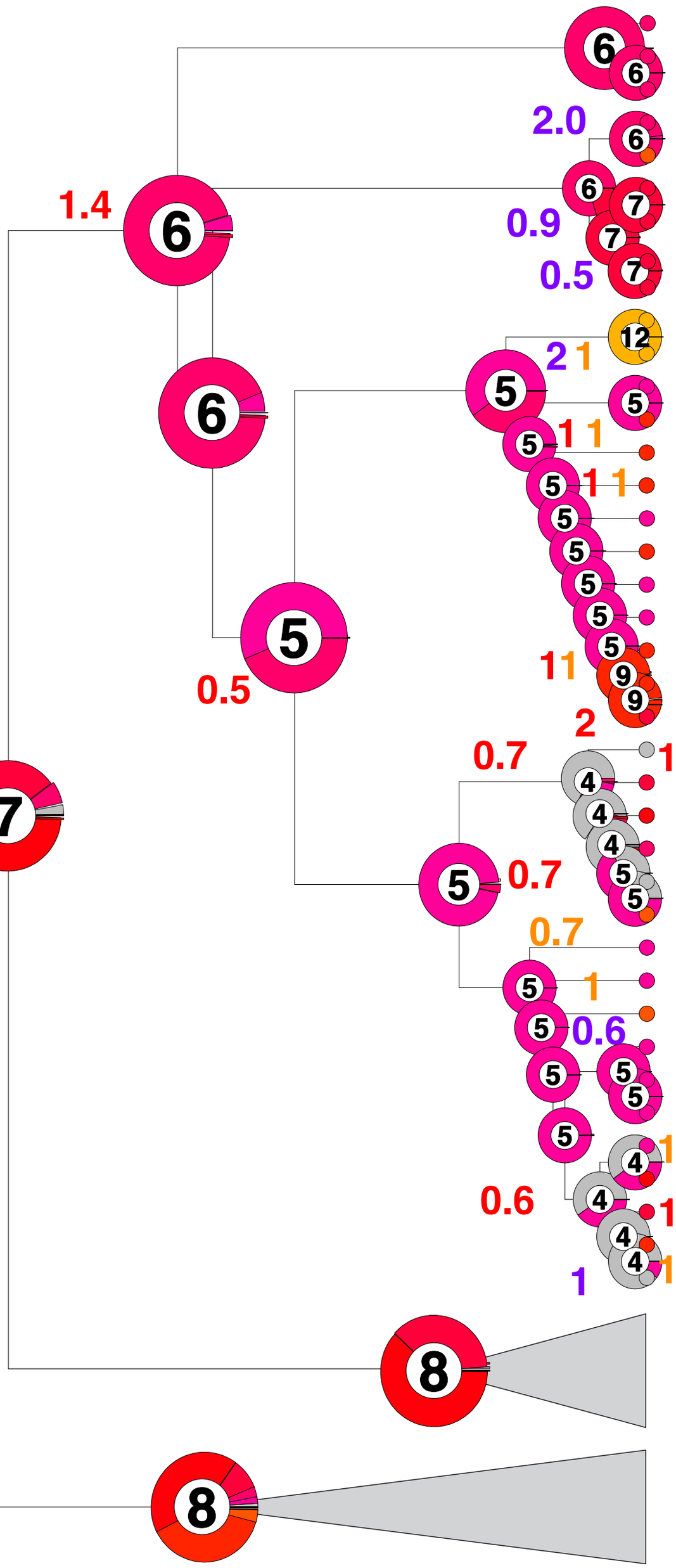

Tulbaghia simmlerii – 6
Tulbaghia capensis – 6
Tulbaghia violacea – 6
Speea humilis – 6
Miersia chilensis – 10
Gilliesia graminea – 7
Solaria atropurpurea – 7
Gilliesia montana – 7
Solaria miersioides – 7
Zoellnerallium andinum – 12
Zoellnerallium serenense – 12
Leucocoryne vittata – 5
Leucocoryne narcissoides – 9
Leucocoryne dimorphopetala – 9
Leucocoryne talinensis – 9
Leucocoryne angustipetala – 5
Leucocoryne ixioides – 9
Leucocoryne coquimbensis – 5
Leucocoryne purpurea – 5
Leucocoryne pauciflora – 9
Leucocoryne appendiculata – 9
Leucocoryne violacescens – 7
Tristagma graminifolium – 4
Ipheion tweedieanum – 7
Tristagma nivale – 8
Ipheion uniflorum – 6
Tristagma bivalve – 4
Ipheion recurvifolium – 10
Nothoscordum nudicaule – 5
Nothoscordum arenarium – 5
Nothoscordum gracile –10
Nothoscordum felipponei – 5
Nothoscordum setaceum – 5
Nothoscordum ostenii – 5
Nothoscordum pulchellum – 5
Nothoscordum montevidense – 8
Nothoscordum bonariense – 7
Nothoscordum bivalve – 9
Nothoscordum gaudichaudianum – 4

Events inferred with an exp. > 0.5: 262.6

Chromosome gains : 93

Chromosome losses : 83.1

Duplications : 86.5

Demiduplications : 0

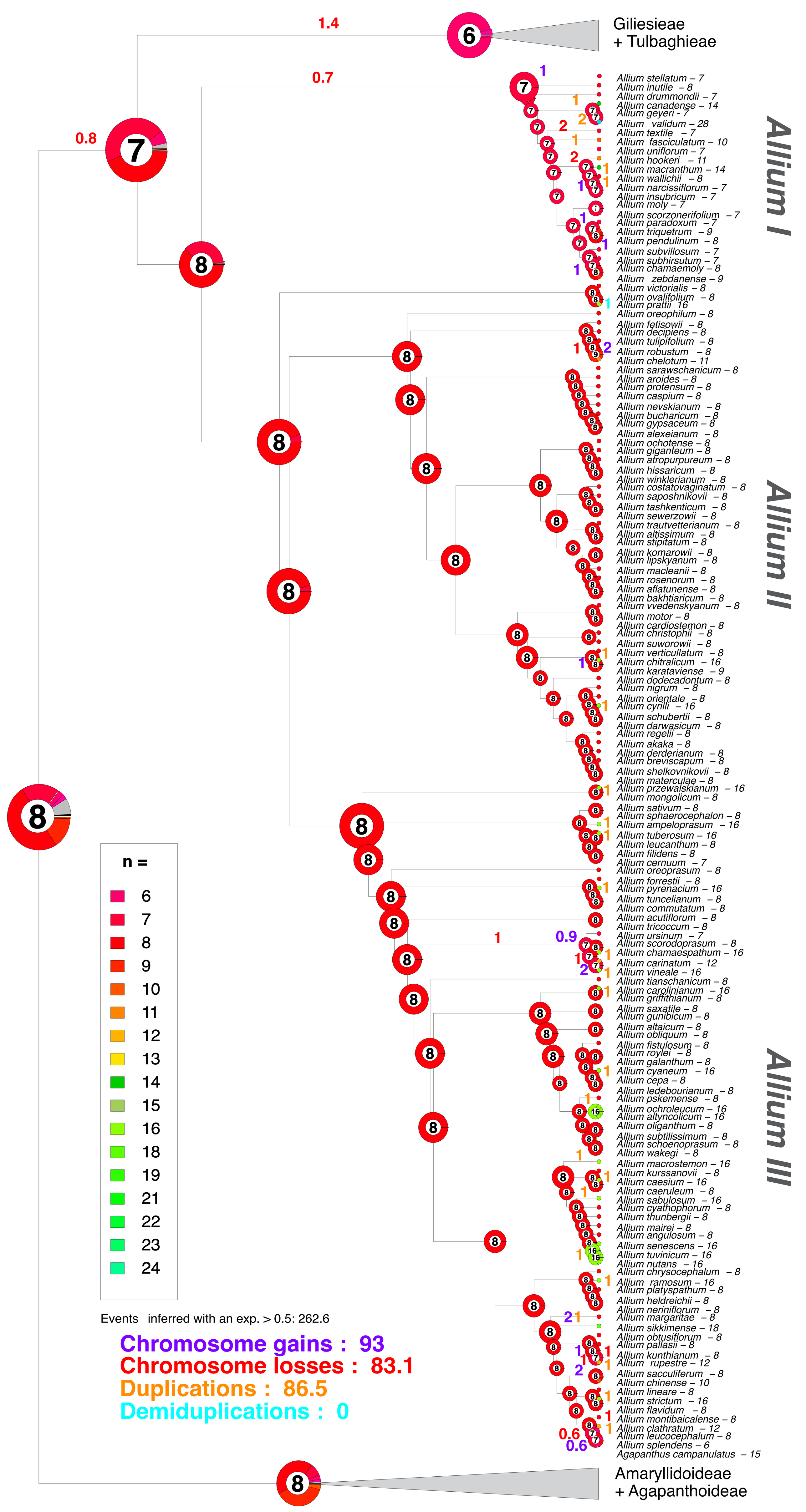
Giliesieae
+ Tulbaghieae
Allium I
Allium II
Allium III
Amaryllidoideae
+ Agapanthoideae
Allium stellatum – 7
Allium inutile – 8
Allium drummondii – 7
Allium canadense – 14
Allium geyeri - 7
Allium validum – 28
Allium textile – 7
Allium fasciculatum – 10
Allium uniflorum – 7
Allium hookeri – 11
Allium macranthum – 14
Allium wallichii – 8
Allium narcissiflorum – 7
Allium insubricum – 7
Allium moly – 7
Allium scorzonerifolium – 7
Allium paradoxum – 7
Allium triquetrum – 9
Allium pendulinum – 8
Allium subvillosum – 7
Allium subhirsutum – 7
Allium chamaemoly – 8
Allium zebdanense – 9
Allium victorialis – 8
Allium ovalifolium – 8
Allium prattii 16
Allium oreophilum – 8
Allium fetisowii – 8
Allium decipiens – 8
Allium tulipifolium – 8
Allium robustum – 8
Allium chelotum – 11
Allium sarawschanicum – 8
Allium aroides – 8
Allium protensum – 8
Allium caspium – 8
Allium nevskianum – 8
Allium bucharicum – 8
Allium gypsaceum – 8
Allium alexeianum – 8
Allium ochotense – 8
Allium giganteum – 8
Allium atropurpureum – 8
Allium hissaricum – 8
Allium winklerianum – 8
Allium costatovaginatum – 8
Allium saposhnikovii – 8
Allium tashkenticum – 8
Allium sewerzowii – 8
Allium trautvetterianum – 8
Allium altissimum – 8
Allium stipitatum – 8
Allium komarowii – 8
Allium lipskyanum – 8
Allium macleanii – 8
Allium rosenorum – 8
Allium aflatunense – 8
Allium bakhtiaricum – 8
Allium vvedenskyanum – 8
Allium motor – 8
Allium cardiostemon – 8
Allium christophii – 8
Allium suworowii – 8
Allium verticullatum – 8
Allium chitralicum – 16
Allium karataviense – 9
Allium dodecadontum – 8
Allium nigrum – 8
Allium orientale – 8
Allium cyrilli – 16
Allium schubertii – 8
Allium darwasicum – 8
Allium regelii – 8
Allium akaka – 8
Allium derderianum – 8
Allium breviscapum – 8
Allium shelkovnikovii – 8
Allium materculae – 8
Allium przewalskianum – 16
Allium mongolicum – 8
Allium sativum – 8
Allium sphaerocephalon – 8
Allium ampeloprasum – 16
Allium tuberosum – 16
Allium leucanthum – 8
Allium filidens – 8
Allium cernuum – 7
Allium oreoprasum – 8
Allium forrestii – 8
Allium pyrenacium – 16
Allium tuncelianum – 8
Allium commutatum – 8
Allium acutiflorum – 8
Allium tricoccum – 8
Allium ursinum – 7
Allium scorodoprasum – 8
Allium chamaespathum – 16
Allium carinatum – 12
Allium vineale – 16
Allium tianschanicum – 8
Allium carolinianum – 16
Allium griffithianum – 8
Allium saxatile – 8
Allium gunibicum – 8
Allium altaicum – 8
Allium obliquum – 8
Allium fistulosum – 8
Allium roylei – 8
Allium galanthum – 8
Allium cyaneum – 16
Allium cepa – 8
Allium ledebourianum – 8
Allium pskemense – 8
Allium ochroleucum – 16
Allium altyncolicum – 16
Allium oliganthum – 8
Allium subtilissimum – 8
Allium schoenoprasum – 8
Allium wakegi – 8
Allium macrostemon – 16
Allium kurssanovii – 8
Allium caesium – 16
Allium caeruleum – 8
Allium sabulosum – 16
Allium cyathophorum – 8
Allium thunbergii – 8
Allium mairei – 8
Allium angulosum – 8
Allium senescens – 16
Allium tuvinicum – 16
Allium nutans – 16
Allium chrysocephalum – 8
Allium ramosum – 16
Allium platyspathum – 8
Allium heldreichii – 8
Allium neriniflorum – 8
Allium margaritae – 8
Allium sikkimense – 18
Allium obtusiflorum – 8
Allium pallasii – 8
Allium kunthianum – 8
Allium rupestre – 12
Allium sacculiferum – 8
Allium chinense – 10
Allium lineare – 8
Allium strictum – 16
Allium flavidum – 8
Allium montibaicalense – 8
Allium clathratum – 12
Allium leucocephalum – 8
Allium splendens – 6
Agapanthus campanulatus – 15
n =
6
7
8
9
10
11
12
13
14
15
16
18
19
21
22
23
24
Events inferred with an exp. > 0.5: 262.6
Chromosome gains : 93
Chromosome losses : 83.1
Duplications : 86.5
Demiduplications : 0

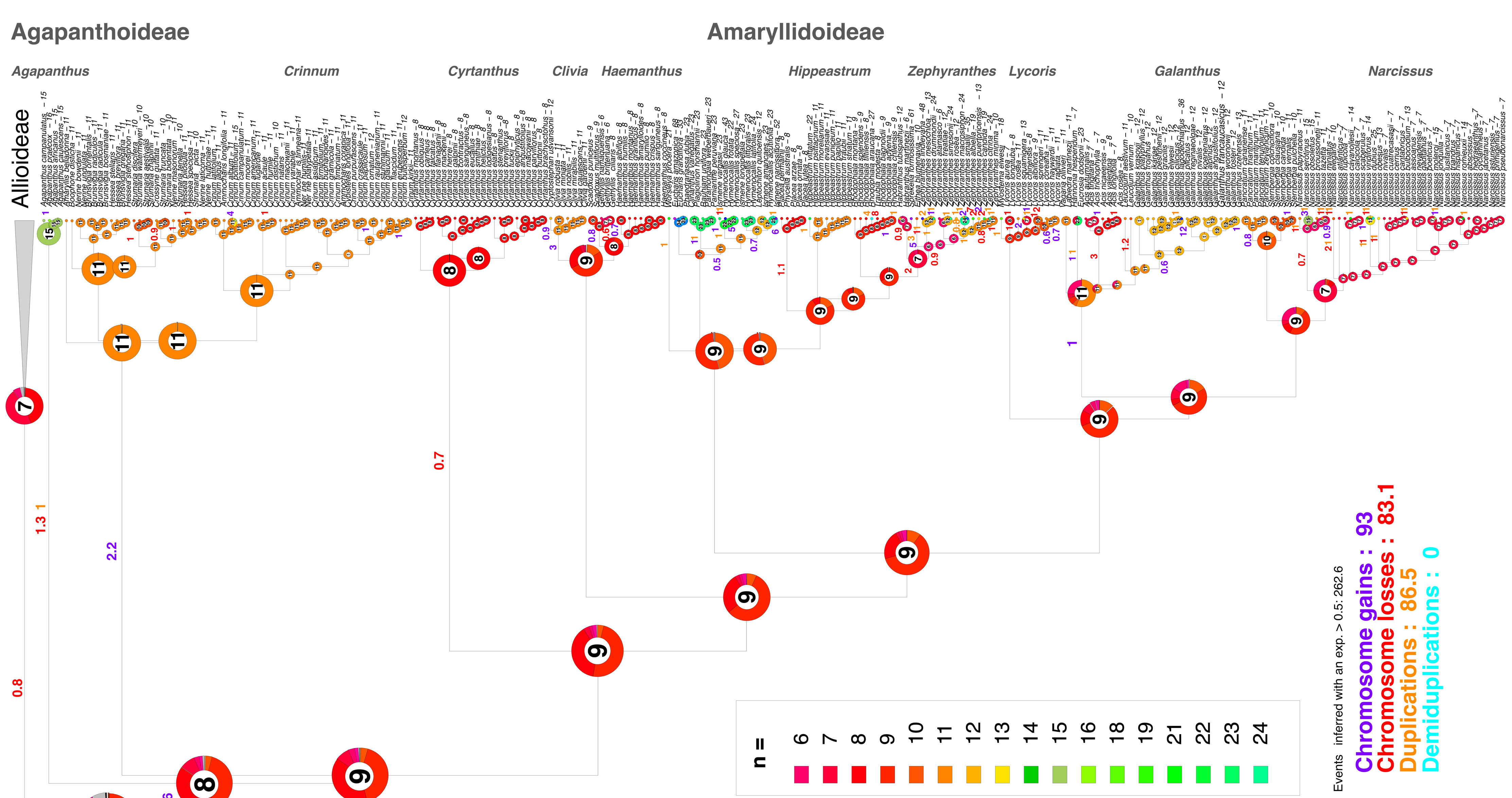
Agapanthoideae
Amaryllidoideae
Allioideae
Agapanthus
Crinnum
Cyrtanthus
Clivia
Haemanthus
Hippeastrum
Zephyranthes
Lycoris
Galanthus
Narcissus
n =
6
7
8
9
10
11
12
13
14
15
16
18
19
21
22
23
24
Events inferred with an exp. > 0.5: 262.6
Chromosome gains : 93
Chromosome losses : 83.1
Duplications : 86.5
Demiduplications : 0
