# Supplementary material for: Divide to Conquer: Evolutionary History of Allioideae Tribes (Amaryllidaceae) Is Linked to Distinct Trends of Karyotype Evolution
Source: Front Plant Sci. 2020 Apr 7;11:320. doi: 10.3389/fpls.2020.00320 (PMC7155398; doi:10.3389/fpls.2020.00320)
Supplement: TABLE S1 — List of Amaryllidaceae species, haploid chromosome numbers (n), genome size of the diploid chromosome complement (2C) in picograms (pg), number of 35S and 5S rDNA sites, references for both 2C and rDNA sites number and GenBank accession numbers for the four regions used in this work. * = 2C values obtained from the Royal Botanic Gardens Kew C-value database (https://cvalues.science.kew.org/, Bennett and Leitch, 2012). [file Table_1.docx]

Supplementary Table 1 - List of Amaryllidaceae species, haploid chromosome numbers (n), genome size of the diploid chromosome complement (2C) in picograms (pg), number of 35S and 5S rDNA sites, references for both 2C and rDNA sites number and GenBank accession numbers for the four regions used in this work. * = 2C values obtained from the Royal Botanic Gardens Kew C-value database (<https://cvalues.science.kew.org/>, Bennett and Leitch 2012)

| **Species** | ***n*** | **2C** | **Ref. 2C** | **rDNA sites** | | **Ref. DNAr** | **ITS** | ***mat*K** | ***ndh*F** | ***rbc*L** |
| --- | --- | --- | --- | --- | --- | --- | --- | --- | --- | --- |
|  |  |  |  | **35S** | **5S** |  |  |  |  |  |
| **Agapanthoideae** |  |  |  |  |  |  |  |  |  |  |
| *Agapanthus africanus* (L.) Hoffmanns. | 15 | 31.6 | * | 2 | 2 | Reis et al. 2016 | - | JQ024934.1 | AF508405.1 | AY465699.1 |
| *Agapanthus campanulatus* F.M.Leight. | 15 | 22.45 | * | - | - | - | JX464256.1 | JX464521.1 | - | - |
| *Agapanthus caulescens* Spreng. | 15 | 24.38 | * | - | - | - | AF373070.1 | - | - | - |
| *Agapanthus praecox* Willd. | 16 | 25.36 | * | - | - | - | - | HQ180852.1 | HQ181102.1 | HQ182414.1 |
| **Allioideae** |  |  |  |  |  |  |  |  |  |  |
| Tribe Allieae |  |  |  |  |  |  |  |  |  |  |
| [*Allium acutiflorum* Loisel.](http://ccdb.tau.ac.il/Angiosperms/Amaryllidaceae/Allium/Allium%20acutiflorum%20Loisel./) | 8 | - | - | - | - | - | EU626389.1 | - | - | - |
| *Allium aflatunense* B.Fedtsch | 8 | 43.71 | Gurushidze et al. 2016 | - | - | - | AF037615.1 | - | - | - |
| *Allium akaka* S.G.Gmel. ex Schult. & Schult.f. | 8 | 39.39 | Gurushidze et al. 2016 | - | - | - | FM177244.1 | - | - | - |
| *Allium alexeianum* Regel | 8 | 34.18 | Gurushidze et al. 2016 | - | - | - | FM177249.1 | - | - | - |
| *Allium altaicum* Pall. | 8 | 29.1 | * | - | - | - | MG282017.1 | MG282043.1 | - | AF206731.1 |
| *Allium altissimum* Regel | 8 | 43.89 | Gurushidze et al. 2016 | - | - | - | FM177252.1 | - | - | - |
| *Allium altyncolicum* N.Friesen | 16 | 35.25 | * | - | - | - | AY427528.1 | - | - | - |
| [*Allium ampeloprasum* L.](http://ccdb.tau.ac.il/Angiosperms/Amaryllidaceae/Allium/Allium%20ampeloprasum%20L./) | 16 | 60 | * | - | - | - | FJ664336.1 | - | - | - |
| *Allium angulosum* L. | 8 | 30.2 | * | - | - | - | LN867002.1 | - | - | - |
| *Allium aroides* Popov & Vved. | 8 | 38.09 | Gurushidze et al. 2016 | - | - | - | FM177258.1 | - | - | - |
| *Allium atropurpureum* Waldst. & Kit. | 8 | 52.24 | Gurushidze et al. 2016 | - | - | - | AF037616.1 | - | - | - |
| *Allium bakhtiaricum* Regel | - | 41.28 | Gurushidze et al. 2016 | - | - | - | FM177269.1 | - | - | - |
| *Allium breviscapum* Stapf | 8 | 45.95 | Gurushidze et al. 2016 | - | - | - | FM177274.1 | - | - | - |
| *Allium bucharicum* Regel | 8 | 28.5 | * | - | - | - | FM177276.1 | - | - | - |
| *Allium caeruleum* Pall. | 8 | 23.45 | * | - | - | - | MG772547.1 | MG772531.1 | - | JX017603.1 |
| *Allium caesium* Schrenk | 16 | 25.85 | * | - | - | - | MG282023.1 | MG282049.1 | - | - |
| [*Allium canadense* L.](http://ccdb.tau.ac.il/Angiosperms/Amaryllidaceae/Allium/Allium%20canadense%20L./) | 14 | 88.5 | * | - | - | - | - | - | - | KJ773255.1 |
| *Allium cardiostemon* Fisch. & C.A.Mey. | 8 | 41.14 | Gurushidze et al. 2016 | - | - | - | AJ411971.1 | - | - | - |
| *Allium carinatum* L. | 12 | 32.65 | * | - | - | - | - | - | - | HE963312.1 |
| *Allium carolinianum* DC. | 16 | 26.45 | * | - | - | - | KT762157.1 | HQ690720.1 | - | HQ690453.1 |
| *Allium caspium* (Pall.) M.Bieb. | 8 | 31.9 | * | - | - | - | MG282008.1 | MG282034.1 | - | - |
| [*Allium cepa* L.](http://ccdb.tau.ac.il/Angiosperms/Amaryllidaceae/Allium/Allium%20cepa%20L./) | 8 | 33.5 | * | 4 | 4 | Mancia et al. 2016 | FJ664287.1 | - | - | JQ273896.1 |
| *Allium cernuum* Roth | 7 | 34.2 | * | - | - | - | KU145489.1 | - | - | KX678878.1 |
| [*Allium chamaespathum* Boiss.](http://ccdb.tau.ac.il/Angiosperms/Amaryllidaceae/Allium/Allium%20chamaespathum%20Boiss./) | 8 | - | - | - | - | - | KP221821.1 | - | - | - |
| *Allium chinense* G.Don | 16 | 65.5 | * | - | - | - | KT781694.1 | - | - | - |
| *Allium chamaemoly* L. | 11 | 28.76 | * | - | - | - | HF934271.1 | - | - | - |
| *Allium chelotum* Wendelbo | 10 | 39.74 | Gurushidze et al. 2016 | - | - | - | FM177465.1 | - | - | - |
| *Allium chitralicum* F.T.Wang & Tang | 16 | 68.69 | Gurushidze et al. 2016 | - | - | - | FM177283.1 | - | - | - |
| [*Allium chrysocephalum* Regel](http://ccdb.tau.ac.il/Angiosperms/Amaryllidaceae/Allium/Allium%20chrysocephalum%20Regel/) | 8 | - | - | - | - | - | - | JF953061.1 | - | - |
| *Allium clathratum* Ledeb. | 8 | 24.35 | * | - | - | - | AJ411855.1 | - | - | - |
| [*Allium commutatum* Guss.](http://ccdb.tau.ac.il/Angiosperms/Amaryllidaceae/Allium/Allium%20commutatum%20Guss./) | 12 | - | - | 2 | 4 | Besendorfer et al. 2002 | EU626388.1 | - | - | - |
| *Allium costatovaginatum* Kamelin & Levichev | - | 34.46 | Gurushidze et al. 2016 | - | - | - | FM177288.1 | - | - | - |
| *Allium cristophii* Trautv. | 8 | 45.96 | Gurushidze et al. 2016 | - | - | - | AF037610.1 | - | - | - |
| [*Allium cyaneum* Regel](http://ccdb.tau.ac.il/Angiosperms/Amaryllidaceae/Allium/Allium%20cyaneum%20Regel/) | 16 | - | - | 3 | 6 | Lee et al. 1998 | - | - | - | HQ690445.1 |
| [*Allium cyathophorum var. farreri* (Stearn) Stearn](http://ccdb.tau.ac.il/Angiosperms/Amaryllidaceae/Allium/Allium%20cyathophorum%20var.%20farreri%20(Stearn)%20Stearn/) | 8 | - | - | - | - | - | KP114588.1 | - | - | - |
| *Allium cyrilli* Ten. | 16 | 78.73 | Gurushidze et al. 2016 | - | - | - | FM177463.1 | - | - | - |
| *Allium darwasicum* Regel | 8 | 33.9 | * | - | - | - | AF037617.1 | - | - | - |
| *Allium decipiens* Fisch. ex Schult. & Schult.f. | 8 | 42.22 | Gurushidze et al. 2016 | - | - | - | FM177307.1 | - | - | - |
| *Allium derderianum* Regel | 8 | 43.42 | Gurushidze et al. 2016 | - | - | - | FM177308.1 | - | - | - |
| *Allium dodecadontum* Vved. | 8 | 37.41 | Gurushidze et al. 2016 | - | - | - | FM177461.1 | - | - | - |
| *Allium drummondii* Regel | 7 | 45.3 | * | - | - | - | AY427534.1 | - | - | - |
| [*Allium fasciculatum* Rendle](http://ccdb.tau.ac.il/Angiosperms/Amaryllidaceae/Allium/Allium%20fasciculatum%20Rendle/) | 10 | - | - | - | - | - | JX017538.1 | - | - | - |
| *Allium fetisowii* Regel | 8 | 26.26 | * | - | - | - | AF037619.1 | - | - | - |
| *Allium filidens* Regel | 8 | 49.45 | * | - | - | - | AJ412723.1 | - | - | - |
| [*Allium fistulosum* L.](http://ccdb.tau.ac.il/Angiosperms/Amaryllidaceae/Allium/Allium%20fistulosum%20L./) | 8 | 25.3 | * | 4 | 2 | Lee et al. 1999 | - | JQ276390.1 | - | JQ273895.1 |
| [*Allium flavidum* Ledeb.](http://ccdb.tau.ac.il/Angiosperms/Amaryllidaceae/Allium/Allium%20flavidum%20Ledeb./) | 8 | - | - | - | - | - | - | JN864809.1 | - | - |
| [*Allium forrestii* Diels](http://ccdb.tau.ac.il/Angiosperms/Amaryllidaceae/Allium/Allium%20forrestii%20Diels/) | 8 | - | - | - | - | - | - | - | - | HQ690444.1 |
| *Allium galanthum* Kar. & Kir. | 8 | 24.4 | * | - | - | - | GQ181101.1 | MG282045.1 | - | HQ690461.1 |
| *Allium geyeri* S.Watson | 7 | 47.65 | * | - | - | - | EU325672.1 | - | - | MG227128.1 |
| *Allium giganteum* Regel | 8 | 42.81 | * | - | - | - | AF037607.1 | - | - | - |
| *Allium griffithianum* Boiss. | 8 | 20.6 | * | - | - | - | AJ411862.2 | - | - | - |
| *Allium gunibicum* Miscz. ex Grossh. | 8 | 31.1 | * | - | - | - | AJ411890.1 | - | - | - |
| *Allium gypsaceum* Popov & Vved. | 8 | 33.82 | Gurushidze et al. 2016 | - | - | - | AF081255.1 | - | - | - |
| *Allium heldreichii* Boiss. | 8 | 32.3 | * | - | - | - | AY427539.1 | - | - | - |
| *Allium hissaricum* Vved. | 8 | 29.4 | * | - | - | - | FM177330.1 | - | - | - |
| *Allium hookeri* Thwaites | 11 | 31.6 | * | - | - | - | KT762155.1 | - | - | - |
| *Allium insubricum* Boiss. & Reut. | 7 | 42.15 | * | - | - | - | HF934290.1 | - | - | - |
| [*Allium inutile*](http://ccdb.tau.ac.il/countsByMatchedName/Nothoscordum%20inutile%20(Makino)%20Kitam.) Makino | 8 | - | - | - | - | - | - | - | - | AB034751.1 |
| *Allium karataviense* Regel | 9 | 42.66 | Gurushidze et al. 2016 | - | - | - | AF037618.1 | - | - | - |
| *Allium komarowii* Lipsky | 8 | 40.66 | Gurushidze et al. 2016 | - | - | - | AJ411967.1 | - | - | - |
| *Allium kunthianum* Vved. | 8 | 35.1 | * | - | - | - | AJ412734.1 | - | - | - |
| *Allium kurssanovii* Popov | 8 | 32.05 | * | - | - | - | AJ311869.1 | - | - | - |
| *Allium ledebourianum* Schult. & Schult.f. | 8 | 17.8 | * | - | - | - | MG282021.1 | MG282047.1 | - | - |
| [*Allium leucanthum* K. Koch](http://ccdb.tau.ac.il/Angiosperms/Amaryllidaceae/Allium/Allium%20leucanthum%20K.%20Koch/) | 8 | - | - | - | - | - | EU626373.1 | - | - | - |
| *Allium leucocephalum* Turcz. ex Ledeb. | 8 | 26.35 | * | - | - | - | AJ412757.1 | - | - | - |
| *Allium lineare* L. | 8 | 26.2 | * | - | - | - | GQ181103.1 | HQ690741.1 | - | HQ690465.1 |
| *Allium lipskyanum* Vved. | 8 | 33.62 | Gurushidze et al. 2016 | - | - | - | FM177350.1 | - | - | - |
| *Allium macleanii* Baker | 8 | 42.63 | Gurushidze et al. 2016 | - | - | - | AF037608.1 | - | - | - |
| [*Allium macranthum* Baker](http://ccdb.tau.ac.il/Angiosperms/Amaryllidaceae/Allium/Allium%20macranthum%20Baker/) | 14 | - | - | - | - | - | JX017557.1 | - | - | HQ690443.1 |
| *Allium macrostemon* Bunge | 16 | 43.25 | * | - | - | - | KF693245.1 | KC704477.1 | - | GQ436307.1 |
| [*Allium mairei* H. Lév.](http://ccdb.tau.ac.il/Angiosperms/Amaryllidaceae/Allium/Allium%20mairei%20H.%20L%C3%A9v./) | 8 | 28.25 | * | - | - | - | HQ690574.1 | - | - | HQ690467.1 |
| *Allium margaritae* B.Fedtsch. | 8 | 31.5 | * | - | - | - | AJ412732.1 | - | - | - |
| *Allium materculae* Bordz. | 8 | 48.6 | * | - | - | - | FM177358.1 | - | - | - |
| *Allium moly* L. | 7 | 49.95 | * | - | - | - | HF934300.1 | - | - | - |
| *Allium mongolicum* Regel | 8 | 28.7 | * | - | - | - | GU565926.1 | - | - | - |
| *Allium montibaicalense* N.Friesen | 8 | 30.65 | * | - | - | - | AJ411871.1 | - | - | - |
| *Allium motor* Kamelin & Levichev | 8 | 36.46 | Gurushidze et al. 2016 | - | - | - | FM177364.1 | - | - | - |
| *Allium narcissiflorum* Vill. | 7 | 43.2 | * | - | - | - | HF934304.1 | - | - | - |
| *Allium neriniflorum* (Herb.) G.Don | 8 | 44.3 | * | - | - | - | GQ412221.1 | - | - | - |
| *Allium nevskianum* Vved. ex Wendelbo | 8 | 36.6 | Gurushidze et al. 2016 | - | - | - | FM177367.1 | - | - | - |
| *Allium nigrum* L. | 8 | 54.81 | Gurushidze et al. 2016 | - | - | - | KP881228.1 | - | - | - |
| [*Allium nutans* L.](http://ccdb.tau.ac.il/Angiosperms/Amaryllidaceae/Allium/Allium%20nutans%20L./) | 16 | 45.25 | * | - | - | - | - | JN864806.1 | - | - |
| *Allium obliquum* L. | 8 | 26.35 | * | - | - | - | MG182351.1 | - | - | - |
| [*Allium obtusiflorum* DC.](http://ccdb.tau.ac.il/Angiosperms/Amaryllidaceae/Allium/Allium%20obtusiflorum%20DC./) | 8 | - | - | - | - | - | KP221816.1 | - | - | - |
| *Allium ochroleucum* W. & K. | 8 | 32.1 | * | - | - | - | AJ412755.1 | - | - | - |
| [*Allium ochotense* Prokh.](http://ccdb.tau.ac.il/Angiosperms/Amaryllidaceae/Allium/Allium%20ochotense%20Prokh./) | 16 | - | - | - | - | - | - | - | JX903322.1 | - |
| *Allium oliganthum* Kar. & Kir. | 8 | 18.6 | * | - | - | - | AJ411835.1 | - | - | - |
| *Allium oreophilum* C.A.Mey. | 8 | 38.8 | * | - | - | - | AJ411931.1 | MG282032.1 | - | - |
| [*Allium oreoprasum* Schrenk](http://ccdb.tau.ac.il/Angiosperms/Amaryllidaceae/Allium/Allium%20oreoprasum%20Schrenk/) | 8 |  | - | - | - | - | - | JN864815.1 | - | - |
| *Allium orientale* Boiss. | 8 | 43.68 | * | - | - | - | FM177376.1 | - | - | - |
| [*Allium ovalifolium* H. -Mazz.](http://ccdb.tau.ac.il/Angiosperms/Amaryllidaceae/Allium/Allium%20ovalifolium%20H.%20-Mazz./) | 8 | - | - | - | - | - | HQ690530.1 | - | - | - |
| *Allium pallasii* Murray | 8 | 19.85 | * | - | - | - | KF693250.1 | HQ690735.1 | - | - |
| *Allium pendulinum* Ten. | 7 | 37.75 | * | - | - | - | HF934321.1 | - | - | - |
| *Allium paradoxum* (M.Bieb.) G.Don | 8 | 53.5 | * | - | - | - | HF934319.1 | - | - | KF997330.1 |
| [*Allium platyspathum* Schrenk](http://ccdb.tau.ac.il/Angiosperms/Amaryllidaceae/Allium/Allium%20platyspathum%20Schrenk/) | 8 | - | - | - | - | - | - | JN864794.1 | - | - |
| [*Allium prattii* C. H. Wright](http://ccdb.tau.ac.il/Angiosperms/Amaryllidaceae/Allium/Allium%20prattii%20C.%20H.%20Wright/) | 16 | - | - | - | - | - | HQ690552.1 | - | - | - |
| *Allium protensum* Wendelbo | 8 | 59.81 | Gurushidze et al. 2016 | - | - | - | AF037609.1 | - | - | - |
| [*Allium przewalskianum* Regel](http://ccdb.tau.ac.il/Angiosperms/Amaryllidaceae/Allium/Allium%20przewalskianum%20Regel/) | 16 | - | - | - | - | - | HQ690568.1 | - | - | - |
| *Allium pskemense* B.Fedtsch. | 8 | 35.1 | * | - | - | - | AJ411907.1 | KX523130.1 | - | - |
| [*Allium pyrenaicum* Costa & Vayr.](http://ccdb.tau.ac.il/Angiosperms/Amaryllidaceae/Allium/Allium%20pyrenaicum%20Costa%20&%20Vayr./) | 16 | 60 | * | - | - | - | EU626384.1 | - | - | - |
| [*Allium ramosum* L.](http://ccdb.tau.ac.il/Angiosperms/Amaryllidaceae/Allium/Allium%20ramosum%20L./) | 16 | 63 | * | - | - | - | EU096168.1 | - | - | - |
| *Allium regelii* Trautv. | 8 | 42.83 | Gurushidze et al. 2016 | - | - | - | AJ411972.1 | - | - | - |
| *Allium robustum* Kar. & Kir. | 8 | 32.14 | Gurushidze et al. 2016 | - | - | - | FM177391.1 | - | - | - |
| *Allium roylei* Stearn | 8 | 30.85 | * | - | - | - | KT762156.1 | KX523129.1 | - | - |
| *Allium rosenorum* R.M.Fritsch | 8 | 40.83 | Gurushidze et al. 2016 | - | - | - | FM177399.1 | - | - | - |
| *Allium rupestre* Steven | 12 | 37.8 | * | - | - | - | AJ412733.1 | - | - | - |
| *Allium sacculiferum* Maxim. | 16 | - | - | - | 14 | Seo et al. 2007 | GQ412233.1 | JF972928.1 | - | KC704754.1 |
| *Allium sabulosum* Steven ex Bunge | 8 | 21.1 | * | - | - | - | MG282024.1 | MG282050.1 | - | - |
| *Allium saposhnikovii* Nikitina | 8 | 60.15 | Gurushidze et al. 2016 | - | - | - | FM177405.1 | - | - | - |
| *Allium sarawschanicum* Regel | 8 | 34.2 | Gurushidze et al. 2016 | - | - | - | AJ411935.1 | - | - | - |
| [*Allium sativum* L.](http://ccdb.tau.ac.il/Angiosperms/Amaryllidaceae/Allium/Allium%20sativum%20L./) | 8 | 32.45 | * | - | 6 | Lee et al. 1999 | EU626375.1 | - | - | - |
| [*Allium saxatile* M. Bieb.](http://ccdb.tau.ac.il/Angiosperms/Amaryllidaceae/Allium/Allium%20saxatile%20M.%20Bieb./) | 8 | 27.5 | * | - | - | - | - | JN864797.1 | - | - |
| *Allium schoenoprasum* L. | 8 | 13.38 | * | 2 | - | Garrido et al. 1994 | KU145490.1 | - | - | JN890785.1 |
| *Allium schubertii* Zucc. | 8 | 31.55 | * | - | - | - | FM177411.1 | - | - | - |
| [*Allium scorodoprasum* L.](http://ccdb.tau.ac.il/Angiosperms/Amaryllidaceae/Allium/Allium%20scorodoprasum%20L./) | 8 | 31.5 | * | - | - | - | EU626395.1 | - | - | KF997327.1 |
| *Allium scorzonerifolium* Desf. ex DC. | 7 | 38.45 | * | - | - | - | HF934331.1 | - | - | - |
| [*Allium senescens* L.](http://ccdb.tau.ac.il/Angiosperms/Amaryllidaceae/Allium/Allium%20senescens%20L./) | 16 | 43.2 | * | - | 6 | Lee et al. 1999 | HQ690569.1 | JF953062.1 | - | - |
| *Allium sewerzowii* Regel | 8 | 56.45 | Gurushidze et al. 2016 | - | - | - | MG282009.1 | MG282035.1 | - | - |
| *Allium shelkovnikovii* Grossh | - | 39.43 | Gurushidze et al. 2016 | - | - | - | FM177418.1 | - | - | - |
| [*Allium sikkimense* Baker](http://ccdb.tau.ac.il/Angiosperms/Amaryllidaceae/Allium/Allium%20sikkimense%20Baker/) | 18 | - | - | - | - | - | HQ690572.1 | - | - | - |
| *Allium sphaerocephalon* L. | 8 | 23.85 | * | - | - | - | KX281968.1 | KF997345.1 | - | KX282524.1 |
| *Allium splendens* Willd. ex Schult. & Schult.f. | 6 | 24.3 | * | - | 6 | Lee et al. 1999 | GQ412241.1 | - | - | - |
| *Allium stellatum* Nutt. ex Ker Gawl. | 7 | 25.5 | * | - | - | - | MG216665.1 | - | - | MG228158.1 |
| *Allium stipitatum* Regel | 8 | 43.93 | Gurushidze et al. 2016 | - | - | - | FM177422.1 | - | - | - |
| [*Allium strictum* Schrad.](http://ccdb.tau.ac.il/Angiosperms/Amaryllidaceae/Allium/Allium%20strictum%20Schrad./) | 16 | - | - | - | - | - | - | JN864818.1 | - | - |
| *Allium subhirsutum* L. | 7 | 35.7 | * | - | - | - | HF934336.1 | - | - | - |
| [*Allium subtilissimum* Ledeb.](http://ccdb.tau.ac.il/Angiosperms/Amaryllidaceae/Allium/Allium%20subtilissimum%20Ledeb./) | 8 | - | - | - | - | - | - | JN864820.1 | - | - |
| *Allium subvillosum* Salzm. ex Schult. & Schult.f. | 7 | 28.8 | * | - | - | - | LN823612.1 | - | - | - |
| *Allium suworowii* Regel | 8 | 37.62 | * | - | - | - | AJ411934.1 | - | - | - |
| *Allium tashkenticum* F.O.Khass. & R.M.Fritsch | 8 | 30.44 | Gurushidze et al. 2016 | - | - | - | FM177437.1 | - | - | - |
| [*Allium textile* A. Nelson & J. F. Macbr.](http://ccdb.tau.ac.il/Angiosperms/Amaryllidaceae/Allium/Allium%20textile%20A.%20Nelson%20&%20J.%20F.%20Macbr./) | 7 | - | - | - | - | - | - | - | AF547000.1 | JX848396.1 |
| [*Allium thunbergii* G. Don](http://ccdb.tau.ac.il/Angiosperms/Amaryllidaceae/Allium/Allium%20thunbergii%20G.%20Don/) | 8 | 26.5 | * | - | 6 | Lee et al. 1999 | - | KC704495.1 | JX903324.1 | - |
| [*Allium tianschanicum* Rupr.](http://ccdb.tau.ac.il/Angiosperms/Amaryllidaceae/Allium/Allium%20tianschanicum%20Rupr./) | 8 | - | - | - | - | - | - | JN864800.1 | - | - |
| *Allium trautvetterianum* Regel | 8 | 41.32 | Gurushidze et al. 2016 | - | - | - | FM177439.1 | - | - | - |
| [*Allium tricoccum* Aiton](http://ccdb.tau.ac.il/Angiosperms/Amaryllidaceae/Allium/Allium%20tricoccum%20Aiton/) | 8 | - | - | - | - | - | - | KJ592839.1 | - | - |
| [*Allium triquetrum* L.](http://ccdb.tau.ac.il/Angiosperms/Amaryllidaceae/Allium/Allium%20triquetrum%20L./) | 9 | 36.3 | * | - | - | - | - | - | - | HM849755.1 |
| [*Allium tuberosum* Rottler ex Spreng.](http://ccdb.tau.ac.il/Angiosperms/Amaryllidaceae/Allium/Allium%20tuberosum%20Rottler%20ex%20Spreng./) | 16 | 64.18 | * | 4 | 8 | Do et al. 1999 | - | - | JX096468.1 | JN969275.1 |
| *Allium tulipifolium* Ledeb. | 8 | 31.79 | Sminorv et al. 2017 | - | - | - | FM177442.1 | - | - | - |
| [*Allium tuncelianum* (Kollmann) Özhatay & B. Mathew & Siraneci](http://ccdb.tau.ac.il/Angiosperms/Amaryllidaceae/Allium/Allium%20tuncelianum%20(Kollmann)%20%C3%96zhatay%20&%20B.%20Mathew%20&%20Siraneci/) | 8 | - | - | - | - | - | EU626394.1 | - | - | - |
| *Allium tuvinicum* (N.Friesen) N.Friesen | 16 | 50 | * | - | - | - | LN867019.1 | - | - | - |
| *Allium unifolium* Kellogg | 7 | 32.7 | * | - | - | - | KC119698.1 | - | - | - |
| [*Allium ursinum* L.](http://ccdb.tau.ac.il/Angiosperms/Amaryllidaceae/Allium/Allium%20ursinum%20L./) | 7 | 60.34 | * | - | - | - | - | - | - | KM360624.1 |
| *Allium validum* S.Watson | 28 | 149 | * | - | - | - | HF934357.1 | - | - | MG225721.1 |
| *Allium verticillatum* Regel | 8 | 33.49 | Gurushidze et al. 2016 | - | - | - | AF081256.1 | - | - | - |
| [*Allium victorialis* L.](http://ccdb.tau.ac.il/Angiosperms/Amaryllidaceae/Allium/Allium%20victorialis%20L./) | 8 | 39.33 | * | 4 | 6 | Seo et al. 1997 | HQ690559.1 | - | - | HM640483.1 |
| *Allium vineale* L. | 16 | 39.07 | * | - | - | - | KP221827.1 | HM850504.1 | - | MG228018.1 |
| *Allium vvedenskyanum* Pavlov | 8 | 33.44 | Gurushidze et al. 2016 | - | - | - | FM177451.1 | - | - | - |
| *Allium wakegi* Araki | 8 | - | - | 3 | 6 | Lee et al. 1999 | KU145492.1 | - | - | - |
| [*Allium wallichii* Kunth](http://ccdb.tau.ac.il/Angiosperms/Amaryllidaceae/Allium/Allium%20wallichii%20Kunth/) | 8 | - | - | - | - | - | JX017597.1 | - | - | HQ690448.1 |
| *Allium winklerianum* Regel | 8 | 32.87 | Gurushidze et al. 2016 | - | - | - | FM177453.1 | - | - | - |
| *Allium zebdanense* Boiss. & Noë | 9 | 25.3 | * | - | - | - | MG182350.1 | - | - | - |
| Tribe Gilliesieae |  |  |  |  |  |  |  |  |  |  |
| *Gilliesia graminea* Lindl. | 7 | 36.04 | This Work | 6 | 2 | This Work | HQ393006.1 | HQ392944.1 | HQ392923.1 | JQ273897.1 |
| *Gilliesia montana* Poepp. & Endl. | 13 | - | - | 6 | 2 | This Work | LT718328.1 | LT718266.1 | LT718390.1 | - |
| *Ipheion recurvifolium* (Wright) Traub | 10 | 18.1 | This Work | 10 | 4 | Souza et al. 2010 | KJ918460.1 | - | - | - |
| *Ipheion tweedieanum* (Baker) Traub | 7 | 22.68 | This Work | 14 | 2 | Souza et al. 2010 | KJ918461.1 | - | - | - |
| [*Ipheion uniflorum* Raf.](http://ccdb.tau.ac.il/Angiosperms/Amaryllidaceae/Ipheion/Ipheion%20uniflorum%20Raf./) | 6 | 18.06 | This Work | 4 | 4 | Souza et al. 2010 | AJ412715.1 | HM640598.1 | HQ392927.1 | HM640484.1 |
| *Leucocoryne angustipetala* Gay | 5 | - | - | - | - | - | KF171092.1 | - | - | - |
| *Leucocoryne appendiculata* Phil. | 9 | - | - | 4 | 8 | Souza et al. 2015 | KF171088.1 | LT718285.1 | LT718409.1 | - |
| [*Leucocoryne coquimbensis* F. Phil. ex Phil.](http://ccdb.tau.ac.il/Angiosperms/Amaryllidaceae/Leucocoryne/Leucocoryne%20coquimbensis%20F.%20Phil.%20ex%20Phil./) | 5 | 56.82 | This Work | 4 | 5 | Souza et al. 2015 | HQ393013.1 | JQ435523.1 | - | - |
| *Leucocoryne dimorphopetala* (Gay) Ravenna | 9 | - | - | 4 | 7 | Souza et al. 2015 | KF171087 | - | - | - |
| [*Leucocoryne ixioides* (Sims) Lindl.](http://ccdb.tau.ac.il/Angiosperms/Amaryllidaceae/Leucocoryne/Leucocoryne%20ixioides%20(Sims)%20Lindl./) | 9 | 109.94 | This Work | 4 | 4 | Souza et al. 2015 | KF171093.1 | HQ392952.1 | - | - |
| [*Leucocoryne narcissoides* Phil.](http://ccdb.tau.ac.il/Angiosperms/Amaryllidaceae/Leucocoryne/Leucocoryne%20narcissoides%20Phil./) | 9 | - | - | 6 | 7 | Souza et al. 2015 | KF171095.1 | HQ392953.1 | HQ392932.1 | - |
| *Leucocoryne pauciflora* Phil | 9 | - | - | - | - | - | HQ393015.1 | HQ392954.1 | HQ392933.1 | AF116998.1 |
| [*Leucocoryne purpurea* Gay](http://ccdb.tau.ac.il/Angiosperms/Amaryllidaceae/Leucocoryne/Leucocoryne%20purpurea%20Gay/) | 5 | 54.47 | This Work | 4 | 2 | Souza et al. 2015 | KF171098.1 | HQ392955.1 | HQ392934.1 | - |
| *Leucocoryne talinensis* Lindl. | 9 | 106.32 | This Work | 4 | 12 | Souza et al. 2015 | KJ918468.1 | - | - | - |
| *Leucocoryne violacescens* Phil. | 7 | - | - | 4 | 4 | Souza et al. 2015 | KF171100.1 | - | - | - |
| *Leucocoryne vittata* Ravenna | 5 | - | - | 4 | 2 | Souza et al. 2015 | LT718372.1 | LT718310.1 | LT718434.1 | - |
| [*Miersia chilensis* Lindl.](http://ccdb.tau.ac.il/Angiosperms/Amaryllidaceae/Miersia/Miersia%20chilensis%20Lindl./) | 10 | 52.92 | Pellicer et al. 2017 | 26 | 6 | This Work | KF171078.1 | HQ392965.1 | - | FN870872.1 |
| *Nothoscordum arenarium* Herter | 5 | - | - | 4 | 2 | Souza et al. 2019 | JN591603.1 | - | - | - |
| [*Nothoscordum bivalve* (L.) Britton](http://ccdb.tau.ac.il/Angiosperms/Amaryllidaceae/Nothoscordum/Nothoscordum%20bivalve%20(L.)%20Britton/) | 9 | - | - | 4 | 10 | Souza et al. 2019 | KF419388.1 | KJ772958.1 | - | JX903138.1 |
| *Nothoscordum bonariense* (Pers.) Beauverd | 7 | 81.49 | Souza et al. 2019 | 4 | 14 | Souza et al. 2016 | KT378156.1 | - | - | - |
| *Nothoscordum felipponei* Beauverd | 5 | 37.34 | This Work | 8 | 8 | Souza et al. 2010 | KJ918457.1 | - | - | - |
| [*Nothoscordum gaudichaudianum* Kunth](http://ccdb.tau.ac.il/Angiosperms/Amaryllidaceae/Nothoscordum/Nothoscordum%20gaudichaudianum%20Kunth/) | 4 | 29.73 | Souza et al. 2019 | 6 | 6 | Souza et al. 2019 | HQ393018.1 | HQ392958.1 | - | HQ392981.1 |
| [*Nothoscordum gracile*](http://ccdb.tau.ac.il/countsByMatchedName/Nothoscordum%20fragrans%20(Vent.)%20Kunth) (Aiton) Stearn | 10 | 92.59 | * | 4 | 4 | Souza et al. 2016a | EU096195.1 | - | - | HM850202.1 |
| [*Nothoscordum montevidense* Beauverd](http://ccdb.tau.ac.il/Angiosperms/Amaryllidaceae/Nothoscordum/Nothoscordum%20montevidense%20Beauverd/) | 8 | 35.54 | Souza et al. 2019 | 6 | 6 | Souza et al. 2016a | HQ393020.1 | HQ392960.1 | HQ392939.1 | HQ392983.1 |
| [*Nothoscordum nudicaule* (Lehm.) Guagl.](http://ccdb.tau.ac.il/Angiosperms/Amaryllidaceae/Nothoscordum/Nothoscordum%20nudicaule%20(Lehm.)%20Guagl./) | 5 | 46.96 | * | 4 | 4 | Souza et al. 2016a | JN591606.1 | HQ392961.1 | - | - |
| *Nothoscordum ostenii* Beauverd | 5 | - | - | 18 | 4 | Souza et al. 2019 | KX811640.1 | - | - | - |
| *Nothoscordum pulchellum* Kunth | 5 | 48.27 | This Work | 2 | 2 | Souza et al. 2019 | KT378160.1 | - | - | - |
| *Nothoscordum setaceum* (Baker) Ravenna | 5 | - | - | - | - | - | KX811637.1 | - | - | - |
| *Solaria atropurpurea* (Phil.) Ravenna | 7 | 36.74 | This Work | 6 | 2 | This Work | HQ393007.1 | LT718263.1 | LT718387.1 | Z69207.1 |
| *Solaria miersioides* Phil. | 15 | 36.82 | Pellicer et al. 2016 | 6 | 2 | This Work | LT718378.1 | LT718316.1 | LT718439.1 | - |
| *Speea humilis* Loes. ex K.Krause | 6 | 56.65 | This work | 2 | 2 | This Work | LT718379.1 | LT718317.1 | - | KY047637.1 |
| [*Tristagma bivalve* (Hook.) Traub](http://ccdb.tau.ac.il/Angiosperms/Amaryllidaceae/Tristagma/Tristagma%20bivalve%20(Hook.)%20Traub/) | 4 | - | - | - | - | - | HQ393023.1 | - | - | - |
| [*Tristagma graminifolium*](http://ccdb.tau.ac.il/countsByMatchedName/Garaventia%20graminifolia%20(Phil.)%20Looser) (Phil.) Ravenna | 4 | - | - | - | - | - | KJ918464.1 | - | - | - |
| [*Tristagma nivale* Poepp.](http://ccdb.tau.ac.il/Angiosperms/Amaryllidaceae/Tristagma/Tristagma%20nivale%20Poepp./) | 8 | - | - | - | - | - | HQ393024.1 | JX903552.1 | HQ392942.1 | JX903141.1 |
| *Zoellnerallium andinum* (Poepp.) Crosa | 12 | - | - | 4 | 16 | Souza et al. 2016b | KJ918467.1 | - | - | - |
| *Zoellnerallium serenense* (Ravenna) Crosa | 12 | 26.47 | This Work | 4 | 16 | Souza et al. 2016b | KJ918463.1 | - | - | - |
| Tribe Tulbaghieae |  |  |  |  |  |  |  |  |  |  |
| [*Tulbaghia capensis* L.](http://ccdb.tau.ac.il/Angiosperms/Amaryllidaceae/Tulbaghia/Tulbaghia%20capensis%20L./) | 6 | - | - | - | - | - | GQ412258.1 | - | - | - |
| [*Tulbaghia simmleri* Beauverd](http://ccdb.tau.ac.il/Angiosperms/Amaryllidaceae/Tulbaghia/Tulbaghia%20simmleri%20Beauverd/) | 6 | 38.77 | Baez et al. In press | - | - | - | KU692180.1 | - | - | JX903143.1 |
| [*Tulbaghia violacea* Harv.](http://ccdb.tau.ac.il/Angiosperms/Amaryllidaceae/Tulbaghia/Tulbaghia%20violacea%20Harv./) | 6 | 39.65 | Baez et al. In press | - | - | - | KT373964.1 | JQ276393.1 | AY225028.1 | JQ273898.1 |
| **Amaryllidoideae** |  |  |  |  |  |  |  |  |  |  |
| [*Acis autumnalis*](https://www.ncbi.nlm.nih.gov/nuccore/AY101284.1) (L.) Sweet | 7 | - | - | - | - | - | AY101284.1 | AY101317.1 | AY434489.1 | - |
| [*Acis longifolia*](https://www.ncbi.nlm.nih.gov/nuccore/AY101286.1) J.Gay ex M.Roem. | 7 | - | - | - | - | - | AY101286.1 | AY101319.1 | - | - |
| [*Acis nicaeensis*](https://www.ncbi.nlm.nih.gov/nuccore/AY751419.1) (Ardoino) Lledó, A.P.Davis & M.B.Crespo | 9 | - | - | - | - | - | AY751419.1 | AY101320.1 | AY747088.1 | - |
| [*Acis rosea*](https://www.ncbi.nlm.nih.gov/nuccore/AY101288.1) (F.Martin bis) Sweet | 8 | - | - | - | - | - | AY101288.1 | JX903568.1 | JQ044758.1 | JX903158.1 |
| [*Acis tingitana*](https://www.ncbi.nlm.nih.gov/nuccore/AY751418.1) (Baker) Lledó, A.P.Davis & M.B.Crespo | 7 | - | - | - | - | - | AY751418.1 | AY101322.1 | AY747089.1 | - |
| [*Acis trichophylla*](https://www.ncbi.nlm.nih.gov/nuccore/AY751417.1) G.Don | 7 | - | - | - | - | - | AY751417.1 | AY101323.1 | JQ044759.1 | - |
| [*Acis valentina*](https://www.ncbi.nlm.nih.gov/nuccore/AY101292.1) (Pau) Lledó, A.P.Davis & M.B.Crespo | 8 | - | - | - | - | - | AY101292.1 | AY101324.1 | - | - |
| [*Amaryllis belladonna*](http://www.ncbi.nlm.nih.gov/nuccore/JX464257.1) L. | 11 | 30.10 | * | - | - | - | JX464257.1 | JX903555.1 | JX903333.1 | HM849767.1 |
| [*Ammocharis coranica*](http://www.ncbi.nlm.nih.gov/nuccore/JX464258.1) (Ker Gawl.) Herb. | 11 | - | - | - | - | - | JX464258.1 | JX464522.1 | FJ264204.1 | - |
| [*Ammocharis longifolia*](http://ccdb.tau.ac.il/Angiosperms/Amaryllidaceae/Ammocharis/Ammocharis%20longifolia%20(L.)%20Herb./) (L.) Herb. | 11 | - | - | - | - | - | - | - | - | AM234803.1 |
| [*Ammocharis tinneana*](http://www.ncbi.nlm.nih.gov/nuccore/KR734233.1) (Kotschy & Peyr.) Milne-Redh. & Schweick. | 11 | - | - | - | - | - | KR734233.1 | - | - | KR737345.1 |
| [*Boophone disticha*](http://www.ncbi.nlm.nih.gov/nuccore/JX464259.1) (L.f.) Herb. | 11 | - | - | - | - | - | JX464259.1 | JX464523.1 | AY434486.1 | AF116945.1 |
| [*Brunsvigia bosmaniae*](http://www.ncbi.nlm.nih.gov/nuccore/JX464260.1) F.M.Leight. | 11 | - | - | - | - | - | JX464260.1 | JX464524.1 | - | - |
| [*Brunsvigia gregaria*](http://www.ncbi.nlm.nih.gov/nuccore/EU543584.1) R.A.Dyer | 11 | - | - | - | - | - | - | EU527792.1 | - | - |
| [*Brunsvigia orientalis*](http://www.ncbi.nlm.nih.gov/nuccore/JX464261.1) (L.) Aiton ex Eckl. | 11 | 23.15 | Smarda et al. 2014 | - | - | - | JX464261.1 | JX464525.1 | - | AM234805.1 |
| [*Brunsvigia radula*](http://www.ncbi.nlm.nih.gov/nuccore/AF373083.1) (Jacq.) W.T.Aiton | 11 | - | - | - | - | - | AF373083.1 | - | - | - |
| [*Brunsvigia radulosa*](http://www.ncbi.nlm.nih.gov/nuccore/JX464262.1) Herb. | 11 | - | - | - | - | - | - | JX464526.1 | - | - |
| [*Clinanthus variegatus*](https://www.ncbi.nlm.nih.gov/nuccore/JX464322.1) (Ruiz & Pav.) Meerow | 23 | - | - | - | - | - | JX464322.1 | JX464599.1 | FJ264208.1 | - |
| *Clivia caulescens* R.A.Dyer | 11 | - | - | 4 | 2 | Ran et al. 2001 | AF324022.1 | EU527794.1 | - | - |
| [*Clivia gardenii*](https://www.ncbi.nlm.nih.gov/nuccore/AF324026.1) Hook. | 11 | 35.54 | * | 2 | 2 | Ran et al. 2001 | AF324026.1 | - | - | - |
| [*Clivia miniata*](https://www.ncbi.nlm.nih.gov/nuccore/HM140798.1) (Lindl.) Bosse | 11 | 35.25 | * | 4 | 2 | Ran et al. 2001 | HM140798.1 | JX464549.1 | - | KC704772.1 |
| *Clivia mirabilis* Rourke | 11 | - | - | 2 | 2 | Murray et al | - | KX038866.1 | - | KX038956.1 |
| [*Clivia nobilis*](https://www.ncbi.nlm.nih.gov/nuccore/HM140799.1) Lindl. | 11 | - | - | 6 | 2 | Ran et al. 2001 | HM140799.1 | JX464550.1 | AY225031.1 | AF116950.1 |
| [*Clivia robusta*](https://www.ncbi.nlm.nih.gov/nuccore/AF324022.1) B.G.Murray & al. | 11 | - | - | 4 | 2 | Ran et al. 2001 | - | KX038877.1 | - | KX038940.1 |
| [*Crinum acaule* Baker](http://ccdb.tau.ac.il/Angiosperms/Amaryllidaceae/Crinum/Crinum%20acaule%20Baker/) | 11 | - | - | - | - | - | AY139118.1 | - | - | - |
| [*Crinum album*](http://www.ncbi.nlm.nih.gov/nuccore/AY139151.1) (Forssk.) Herb. | 11 | - | - | - | - | - | AY139151.1 | - | AY225032.1 | - |
| *Crinum americanum* L. | 11 | - | - | 2 | 4 | Santos et al. 2014 | AY139119.1 | KJ772682.1 | - | - |
| *Crinum asiaticum* L. | 11 | 50.5 | * | 2 | 4 | Santos et al. 2014 | AY139120.1 | JQ276395.1 | JX903337.1 | KC704776.1 |
| [*Crinum bulbispermum*](http://www.ncbi.nlm.nih.gov/nuccore/AY139123.1) (Burm.f.) Milne-Redh. & Schweick. | 12 | 40.2 | * | - | - | - | AY139123.1 | JX464527.1 | - | - |
| [*Crinum buphanoides*](http://www.ncbi.nlm.nih.gov/nuccore/AY139124.1) Welw. ex Baker | 11 | - | - | - | - | - | AY139124.1 | EU527805.1 | - | - |
| [*Crinum campanulatum*](http://www.ncbi.nlm.nih.gov/nuccore/AF373088.1) Herb. | 11 | - | - | - | - | - | AF373088.1 | EU527806.1 | - | - |
| [*Crinum crassicaule*](http://www.ncbi.nlm.nih.gov/nuccore/AY139126.1) Baker | 11 | - | - | - | - | - | AY139126.1 | - | - | - |
| [*Crinum distichum*](http://www.ncbi.nlm.nih.gov/nuccore/AY139129.1) Herb. | 10 | - | - | - | - | - | AY139129.1 | - | - | - |
| [*Crinum erubescens*](http://www.ncbi.nlm.nih.gov/nuccore/AY139130.1) L.f. ex Aiton | 11 | - | - | - | - | - | AY139130.1 | - | - | - |
| [*Crinum fimbriatulum*](http://www.ncbi.nlm.nih.gov/nuccore/AY139131.1) Baker | 11 | - | - | - | - | - | AY139131.1 | - | - | - |
| [*Crinum firmifolium*](http://www.ncbi.nlm.nih.gov/nuccore/EU836632.1) Baker | 11 | - | - | - | - | - | EU836632.1 | - | - | - |
| [*Crinum flaccidum*](http://www.ncbi.nlm.nih.gov/nuccore/AY139132.1) Herb. | 11 | - | - | - | - | - | AY139132.1 | - | - | - |
| [*Crinum glaucum*](http://www.ncbi.nlm.nih.gov/nuccore/EF111007.1) A.Chev. | 11 | - | - | - | - | - | EF111007.1 | - | - | - |
| [*Crinum graminicola*](http://www.ncbi.nlm.nih.gov/nuccore/EU543595.1) I.Verd. | 11 | - | - | - | - | - | - | EU527810.1 | - | - |
| [*Crinum humile*](http://www.ncbi.nlm.nih.gov/nuccore/AY139134.1) Herb. | 11 | - | - | - | - | - | AY139134.1 | - | - | - |
| [*Crinum jagus*](http://www.ncbi.nlm.nih.gov/nuccore/AY139135.1) (J.Thomps.) Dandy | 11 | - | - | - | - | - | AY139135.1 | - | - | - |
| [*Crinum kirkii*](http://www.ncbi.nlm.nih.gov/nuccore/AY139136.1) Baker | 11 | - | - | - | - | - | AY139136.1 | - | - | - |
| [*Crinum latifolium*](http://www.ncbi.nlm.nih.gov/nuccore/AY139137.1) L. | 11 | - | - | 4 | 11 | Alam et al. 2010 | AY139137.1 | - | - | - |
| [*Crinum lugardiae*](http://www.ncbi.nlm.nih.gov/nuccore/JX464264.1) N.E.Br. | 11 | - | - | - | - | - | JX464264.1 | JX464528.1 | - | - |
| [*Crinum macowanii*](http://www.ncbi.nlm.nih.gov/nuccore/AF373094.1) Baker | 11 | - | - | - | - | - | AF373094.1 | - | - | KR736835.1 |
| [*Crinum moorei*](http://www.ncbi.nlm.nih.gov/nuccore/AY139141.1) Hook.f. | 11 | 41.5 | - | - | - | - | AY139141.1 | JX464529.1 | - | - |
| [*Crinum natans*](http://www.ncbi.nlm.nih.gov/nuccore/EU836636.1) Baker | 11 | 41.9 | - | - | - | - | EU836636.1 | - | - | - |
| [*Crinum ornatum*](http://www.ncbi.nlm.nih.gov/nuccore/DQ386443.1) (Aiton) Herb. | 12 | 29.73 | * | 2 | 2 | Santos et al. 2014 | DQ386443.1 | - | - | - |
| [*Crinum papillosum*](http://www.ncbi.nlm.nih.gov/nuccore/DQ386444.1) Nordal | 11 | - | - | - | - | - | DQ386444.1 | - | - | - |
| [*Crinum politifolium*](http://www.ncbi.nlm.nih.gov/nuccore/DQ386445.1) R.Wahlstr. | 15 | - | - | - | - | - | DQ386445.1 | - | - | - |
| [*Crinum purpurascens*](http://www.ncbi.nlm.nih.gov/nuccore/DQ386446.1) Herb. | 11 | - | - | - | - | - | DQ386446.1 | - | - | - |
| [*Crinum rautanenianum*](http://www.ncbi.nlm.nih.gov/nuccore/DQ386447.1)Schinz | 11 | - | - | - | - | - | DQ386447.1 | - | - | - |
| [*Crinum stuhlmannii*](http://www.ncbi.nlm.nih.gov/nuccore/JX464267.1) Baker | 11 | - | - | - | - | - | JX464267.1 | JX464531.1 | - | - |
| [*Crinum variabile*](http://www.ncbi.nlm.nih.gov/nuccore/AF373090.1) (Jacq.) Herb. | 11 | - | - | - | - | - | AF373090.1 | - | - | - |
| *Crossyne guttata* (L.) D.Müll.-Doblies & U.Müll.-Doblies | 11 | - | - | - | - | - | AF373089.1 | - | FJ264211.1 | - |
| *Cryptostephanus vansonii* Verd. | 12 | - | - | - | - | - | AY280351.1 | JX464551.1 | AY434490.1 | AF116952.1 |
| [*Cybistetes longifolia*](http://www.ncbi.nlm.nih.gov/nuccore/AF373093.1) (L.) Milne-Redh. & Schweick. | 11 | - | - | - | - | - | AF373093.1 | JX903558.1 | FJ264213.1 | JX903148.1 |
| [*Cyrtanthus angustifolius*](http://www.ncbi.nlm.nih.gov/nuccore/GQ160819.1) (L.f.) Aiton | 8 | - | - | - | - | - | GQ160819.1 | - | GQ160860.1 | - |
| [*Cyrtanthus brachyscyphus*](http://www.ncbi.nlm.nih.gov/nuccore/GQ160822.1) Baker | 8 | - | - | - | - | - | GQ160822.1 | EU527820.1 | GQ160863.1 | - |
| [*Cyrtanthus breviflorus*](http://www.ncbi.nlm.nih.gov/nuccore/GQ160823.1) Harv. | 8 | - | - | - | - | - | GQ160823.1 | EU527821.1 | GQ160864.1 | - |
| [*Cyrtanthus carneus*](http://www.ncbi.nlm.nih.gov/nuccore/GQ160824.1) Lindl. | 8 | - | - | - | - | - | GQ160824.1 | - | GQ160865.1 | - |
| [*Cyrtanthus contractus*](http://www.ncbi.nlm.nih.gov/nuccore/GQ160826.1) N.E.Br. | 8 | - | - | - | - | - | GQ160826.1 | JX464545.1 | GQ160867.1 | - |
| [*Cyrtanthus elatus*](http://www.ncbi.nlm.nih.gov/nuccore/GQ160828.1) (Jacq.) Traub | 8 | 29.5 | * | - | - | - | GQ160828.1 | JX903559.1 | FJ264214.1 | JX903149.1 |
| [*Cyrtanthus epiphyticus*](http://www.ncbi.nlm.nih.gov/nuccore/GQ160829.1) J.M.Wood | 8 | - | - | - | - | - | GQ160829.1 | - | GQ160869.1 | - |
| [*Cyrtanthus eucallus*](http://www.ncbi.nlm.nih.gov/nuccore/GQ160830.1) R.A.Dyer | 8 | - | - | - | - | - | GQ160830.1 | EU527825.1 | GQ160870.1 | - |
| [*Cyrtanthus falcatus*](http://www.ncbi.nlm.nih.gov/nuccore/GQ160831.1) R.A.Dyer | 8 | - | - | - | - | - | GQ160831.1 | - | GQ160871.1 | - |
| [*Cyrtanthus flanaganii*](http://www.ncbi.nlm.nih.gov/nuccore/GQ160834.1) Baker | 8 | - | - | - | - | - | GQ160834.1 | - | GQ160874.1 | - |
| [*Cyrtanthus galpinii*](http://www.ncbi.nlm.nih.gov/nuccore/GQ160835.1) Baker | 8 | - | - | - | - | - | GQ160835.1 | - | GQ160875.1 | - |
| [*Cyrtanthus helictus*](http://www.ncbi.nlm.nih.gov/nuccore/GQ160837.1)Lehm. | 8 | - | - | - | - | - | GQ160837.1 | - | GQ160877.1 | - |
| [*Cyrtanthus herrei*](http://www.ncbi.nlm.nih.gov/nuccore/JX464282.1) (F.M.Leight.) R.A.Dyer | 8 | - | - | - | - | - | JX464282.1 | JX464546.1 | AY434484.1 | HE565517.1 |
| [*Cyrtanthus huttonii*](http://www.ncbi.nlm.nih.gov/nuccore/GQ160838.1) Baker | 8 | - | - | - | - | - | GQ160838.1 | - | GQ160878.1 | - |
| [*Cyrtanthus loddigesianus*](http://www.ncbi.nlm.nih.gov/nuccore/GQ160842.1) (Herb.) R.A.Dyer | 8 | - | - | - | - | - | GQ160842.1 | EU527830.1 | GQ160882.1 | - |
| [*Cyrtanthus mackenii*](http://www.ncbi.nlm.nih.gov/nuccore/GQ160843.1) Hook.f. | 8 | - | - | - | - | - | GQ160843.1 | JX464547.1 | - | - |
| [*Cyrtanthus macowanii*](http://www.ncbi.nlm.nih.gov/nuccore/GQ160846.1) Baker | 8 | - | - | - | - | - | GQ160846.1 | - | GQ160886.1 | - |
| [*Cyrtanthus montanus*](http://www.ncbi.nlm.nih.gov/nuccore/GQ160847.1) R.A.Dyer | 8 | 27.7 | * | - | - | - | GQ160847.1 | - | GQ160887.1 | - |
| [*Cyrtanthus obliquus*](http://www.ncbi.nlm.nih.gov/nuccore/GQ160848.1) (L.f.) Aiton | 8 | - | - | - | - | - | GQ160848.1 | - | GQ160888.1 | - |
| [*Cyrtanthus parviflorus*](http://www.ncbi.nlm.nih.gov/nuccore/GQ160850.1) Baker | 8 | - | - | - | - | - | GQ160850.1 | - | GQ160890.1 | - |
| [*Cyrtanthus sanguineus*](http://www.ncbi.nlm.nih.gov/nuccore/GQ160851.1) (Lindl.) Walp. | 8 | - | - | - | - | - | GQ160851.1 | - | GQ160891.1 | - |
| [*Cyrtanthus smithiae*](http://www.ncbi.nlm.nih.gov/nuccore/GQ160852.1) Watt ex Harv. | 8 | - | - | - | - | - | GQ160852.1 | - | GQ160892.1 | - |
| [*Cyrtanthus stenanthus*](http://www.ncbi.nlm.nih.gov/nuccore/GQ160855.1) Baker | 8 | - | - | - | - | - | GQ160855.1 | - | GQ160895.1 | - |
| [*Cyrtanthus tuckii*](http://www.ncbi.nlm.nih.gov/nuccore/GQ160857.1) Baker | 8 | - | - | - | - | - | GQ160857.1 | - | GQ160897.1 | - |
| [*Eithea blumenavia*](https://www.ncbi.nlm.nih.gov/nuccore/KC207421.1) (K.Koch & C.D.Bouché ex Carrière) Ravenna | 10 | - | - | - | - | - | KC207421.1 | JX464576.1 | FJ264215.1 | - |
| [*Eucharis amazonica*](https://www.ncbi.nlm.nih.gov/nuccore/JX464323.1) Linden ex Planch. | 68 | 24.46 | * | - | - | - | JX464323.1 | JX464600.1 | - | - |
| *Eucharis grandiflora* Planch. & Linden |  | 32.6 | * | - | - | - | - | - | - | JQ273901.1 |
| *Eucrosia dodsonii* Meerow & Dehgan | 23 | - | - | - | - | - | AF223544.1 | - | - | - |
| *Galanthus alpinus* Sosn. | 12 | 62.2 | * | - | - | - | JN792413.1 | - | JQ044736.1 | - |
| [*Galanthus angustifolius*](https://www.ncbi.nlm.nih.gov/nuccore/KF181588.1) Koss | 12 | - | - | - | - | - | KF181588.1 | KF181591.1 | KF181597.1 | - |
| [*Galanthus cilicicus*](https://www.ncbi.nlm.nih.gov/nuccore/AY101295.1) Baker | 12 | 65.7 | * | - | - | - | AY101295.1 | AY101328.1 | - | - |
| [*Galanthus elwesii*](https://www.ncbi.nlm.nih.gov/nuccore/JN792420.1) Hook.f. | 12 | 55.3 | * | - | - | - | JN792420.1 | JQ044723.1 | JQ044737.1 | - |
| *Galanthus fosteri* Baker | 12 | 55 | * | - | - | - | AY101297.1 | AY101330.1 | - | - |
| *Galanthus gracilis* Celak*.* | 12 | 54.7 | * | - | - | - | GU329663.1 | JQ044724.1 | - | - |
| [*Galanthus ikariae*](https://www.ncbi.nlm.nih.gov/nuccore/JN792422.1) Baker | 12 | 68.7 | * | - | - | - | JN792422.1 | JQ044725.1 | AY747080.1 | - |
| [*Galanthus koenenianus*](https://www.ncbi.nlm.nih.gov/nuccore/FN663891.1) Lobin, C.D.Brickell & A.P.Davis | 12 | 57.2 | * | - | - | - | FN663872.1 | FN663891.1 | JQ044742.1 | - |
| [*Galanthus krasnovii*](https://www.ncbi.nlm.nih.gov/nuccore/JN792424.1) Khokhr. | 12 | 90.3 | * | - | - | - | JN792424.1 | JQ044727.1 | - | - |
| [*Galanthus lagodechianus*](https://www.ncbi.nlm.nih.gov/nuccore/AY101301.1) Kem.-Nath. | 36 | 164.3 | * | - | - | - | AY101301.1 | FN663892.1 | JQ044745.1 | - |
| [*Galanthus nivalis*](https://www.ncbi.nlm.nih.gov/nuccore/JN792430.1) L. | 12 | 72.2 | * | - | - | - | JN792430.1 | JQ044728.1 | AY747081.1 | KM360794.1 |
| [*Galanthus peshmenii*](https://www.ncbi.nlm.nih.gov/nuccore/JN792431.1) A.P.Davis & C.D.Brickell | 12 | 56.4 | * | - | - | - | JN792431.1 | JQ044729.1 | AY434492.1 | - |
| [*Galanthus platyphyllus*](https://www.ncbi.nlm.nih.gov/nuccore/JN792432.1) Traub & Moldenke | 12 | 90.4 | * | - | - | - | JN792432.1 | JQ044730.1 | JQ044749.1 | - |
| [*Galanthus plicatus*](https://www.ncbi.nlm.nih.gov/nuccore/JN792434.1) | 12 | 55.4 | * | - | - | - | JN792434.1 | JQ044731.1 | AY747083.1 | - |
| [*Galanthus reginae-olgae*](https://www.ncbi.nlm.nih.gov/nuccore/JN792436.1) | 12 | 70.1 | * | - | - | - | JN792436.1 | AY101339.1 | AY747084.1 | - |
| *Galanthus rizehensis Stern* | 13 | 48.5 | * | - | - | - | GU329704.1 | - | - | - |
| [*Galanthus transcaucasicus*](https://www.ncbi.nlm.nih.gov/nuccore/JN792414.1) M.Bieb. | 12 | 81.9 | * | - | - | - | JN792414.1 | AY101340.1 | KF181598.1 | - |
| [*Galanthus trojanus*](https://www.ncbi.nlm.nih.gov/nuccore/JN792437.1) A.P.Davis & Özhatay | 12 | 56.9 | * | - | - | - | JN792437.1 | - | JQ044755.1 | - |
| [*Galanthus woronowii*](https://www.ncbi.nlm.nih.gov/nuccore/AY751433.1) Losinsk. | 12 | 56.3 | * | - | - | - | AY751433.1 | AY101341.1 | AY747085.1 | - |
| [*Gethyllis britteniana*](https://www.ncbi.nlm.nih.gov/nuccore/AY280352.1) Baker | 6 | 46.85 | * | - | - | - | AY280352.1 | JX903561.1 | JX903341.1 | JX903151.1 |
| [*Gethyllis ciliaris*](https://www.ncbi.nlm.nih.gov/nuccore/AY280353.1) (Thunb.) Thunb. | 8 | - | - | - | - | - | AY280353.1 | - | AY434491.1 | AF116957.1 |
| [*Gethyllis namaquensis*](https://www.ncbi.nlm.nih.gov/nuccore/EU527838.1) (Schönland) Oberm. | 6 | - | - | - | - | - | - | EU527838.1 | - | - |
| [*Habranthus martinezii*](https://www.ncbi.nlm.nih.gov/nuccore/JX464299.1) Ravenna | 6 | - | - | - | - | - | JX464299.1 | JX464578.1 | FJ264218.1 | AF116959.1 |
| [*Habranthus tubispathus*](https://www.ncbi.nlm.nih.gov/nuccore/JX464301.1) (L'Hér.) Traub | 12 | - | - | - | - | - | JX464301.1 | JX464580.1 | KC217385.1 | - |
| [*Haemanthus albiflos*](https://www.ncbi.nlm.nih.gov/nuccore/HM140803.1) Jacq. | 8 | 66.58 | Smarda et al. 2014 | - | - | - | HM140803.1 | JX903563.1 | - | JX903153.1 |
| [*Haemanthus amarylloides*](https://www.ncbi.nlm.nih.gov/nuccore/HM140804.1) Jacq. | 8 | - | - | - | - | - | HM140804.1 | JX464554.1 | - | - |
| [*Haemanthus coccineus*](https://www.ncbi.nlm.nih.gov/nuccore/HM140805.1) L. | 8 | - | - | - | - | - | HM140805.1 | JX464555.1 | - | JX572662.1 |
| [*Haemanthus crispus*](https://www.ncbi.nlm.nih.gov/nuccore/EU527841.1) Snijman | 8 | - | - | - | - | - | - | EU527841.1 | - | - |
| [*Haemanthus graniticus*](https://www.ncbi.nlm.nih.gov/nuccore/AY280357.1) Snijman | 8 | - | - | - | - | - | AY280357.1 | - | - | - |
| [*Haemanthus humilis*](https://www.ncbi.nlm.nih.gov/nuccore/HM140808.1) Jacq. | 8 | - | - | - | - | - | HM140808.1 | JX464557.1 | - | AF116960.1 |
| [*Haemanthus montanus*](https://www.ncbi.nlm.nih.gov/nuccore/HM140809.1) Baker | 8 | - | - | - | - | - | HM140809.1 | JX464558.1 | - | - |
| [*Haemanthus pumilio*](https://www.ncbi.nlm.nih.gov/nuccore/AY280358.1) Jacq. | 8 | - | - | - | - | - | AY280358.1 | - | - | - |
| [*Haemanthus sanguineus*](https://www.ncbi.nlm.nih.gov/nuccore/HM140811.1) Jacq. | 8 | - | - | - | - | - | HM140811.1 | JX464559.1 | - | - |
| [*Hannonia hesperidum*](https://www.ncbi.nlm.nih.gov/nuccore/HM010960.1) Braun-Blanq. & Maire | 7 | - | - | - | - | - | HM010960.1 | HM011041.1 | AY747086.1 | AF116961.1 |
| [*Hessea breviflora*](http://www.ncbi.nlm.nih.gov/nuccore/AF373095.1) Herb. | 11 | - | - | - | - | - | AF373095.1 | - | FJ264222.1 | - |
| [*Hessea pilosula*](http://www.ncbi.nlm.nih.gov/nuccore/JX464269.1) D.Müll.-Doblies & U.Müll.-Doblies | 11 | - | - | - | - | - | JX464269.1 | JX464533.1 | - | - |
| [*Hessea speciosa*](http://www.ncbi.nlm.nih.gov/nuccore/JX464270.1) Snijman | 11 | - | - | - | - | - | JX464270.1 | JX464534.1 | - | - |
| [*Hessea stellaris*](http://www.ncbi.nlm.nih.gov/nuccore/AF373096.1) (Jacq.) Herb. | 11 | - | - | - | - | - | AF373096.1 | - | - | - |
| [*Hessea stenosiphon*](http://www.ncbi.nlm.nih.gov/nuccore/AF373087.1) (Snijman) D.Müll.-Doblies & U.Müll.-Doblies | 11 | - | - | - | - | - | AF373087.1 | - | - | - |
| [*Hippeastrum evansiae*](https://www.ncbi.nlm.nih.gov/nuccore/KC207433.1) (Traub & I.S.Nelson) H.E.Moore | 11 | 30.92 | Poggio et al. 2014 | - | - | - | KC207433.1 | - | KC217389.1 | - |
| [*Hippeastrum morelianum*](https://www.ncbi.nlm.nih.gov/nuccore/KC207435.1) Lem. | 11 | 26.8 | Poggio et al. 2014 | - | - | - | KC207435.1 | - | KC217390.1 | - |
| [*Hippeastrum papilio*](https://www.ncbi.nlm.nih.gov/nuccore/JX464304.1)(Ravenna) Van Scheepen | 11 | 29.1 | * | - | - | - | JX464304.1 | JX464583.1 | AY434475.1 | AF116964.1 |
| [*Hippeastrum psittacinum*](https://www.ncbi.nlm.nih.gov/nuccore/KC207437.1) (Ker Gawl.) Herb. | 11 | 31.34 | Poggio et al. 2014 | - | - | - | KC207437.1 | JX903565.1 | KC217392.1 | JX903155.1 |
| [*Hippeastrum puniceum*](https://www.ncbi.nlm.nih.gov/nuccore/KC207438.1) (Lam.) Voss | 11 | 38.69 | Poggio et al. 2014 | - | - | - | KC207438.1 | JX464584.1 | KC217393.1 | KC704777.1 |
| *Hippeastrum striatum* (Lam.) H.E.Moore | 11 | - | - | 2 | 2 | Santos et al. 2014 | KC207439.1 | - | KC217394.1 | - |
| [*Hippeastrum traubii*](https://www.ncbi.nlm.nih.gov/nuccore/KC207440.1) (Moldenke) H.E.Moore | 11 | - | - | - | - | - | KC207440.1 | - | KC217395.1 | - |
| [*Hippeastrum vittatum*](https://www.ncbi.nlm.nih.gov/nuccore/JX464307.1)(L'Hér.) Herb. | 22 | - | - | - | - | - | JX464307.1 | JX464585.1 | - | - |
| [*Hymenocallis acutifolia*](https://www.ncbi.nlm.nih.gov/nuccore/AF223514.1) (Herb. ex Sims) Sweet | 23 | - | - | - | - | - | AF223514.1 | - | - | - |
| [*Hymenocallis glauca*](https://www.ncbi.nlm.nih.gov/nuccore/AF223515.1) (Zucc.) M.Roem. | 43 | - | - | - | - | - | AF223515.1 | - | - | - |
| [*Hymenocallis latifolia*](https://www.ncbi.nlm.nih.gov/nuccore/JX464317.1) (Mill.) M.Roem. | 24 | - | - | - | - | - | JX464317.1 | JX464594.1 | FJ264224.1 | AF116965.1 |
| [*Hymenocallis littoralis*](https://www.ncbi.nlm.nih.gov/nuccore/JX464318.1) (Jacq.) Salisb. | 23 | - | - | - | - | - | JX464318.1 | JX464595.1 | JX903346.1 | AY460395.1 |
| [*Hymenocallis rotata*](https://www.ncbi.nlm.nih.gov/nuccore/JX464320.1) (Ker Gawl.) Herb. | 22 | - | - | - | - | - | JX464320.1 | JX464597.1 | - | - |
| [*Hymenocallis speciosa*](https://www.ncbi.nlm.nih.gov/nuccore/AF223512.1) (L.f. ex Salisb.) Salisb. | 27 | - | - | - | - | - | AF223512.1 | - | - | - |
| [*Ismene amancaes*](http://ccdb.tau.ac.il/countsByMatchedName/Hymenocallis%20amancaes%20(Ruiz%20&%20Pav.)%20G.%20Nicholson) (Ruiz & Pav.) Herb. | 23 | - | - | - | - | - | AF411080.1 | - | - | - |
| [*Ismene longipetala*](http://ccdb.tau.ac.il/countsByMatchedName/Elisena%20longipetala%20Lindl.) (Lindl.) Meerow | 23 | - | - | - | - | - | AF223520.1 | FR832785.1 | - | AF116966.1 |
| *Ismene narcissiflora* (Jacq.) M.Roem. | 52 | - | - | - | - | - | AF223518.1 | - | AY225037.1 | AF116967.1 |
| *Ismene vargasii* (Velarde) Gereau & Meerow | 23 | - | - | - | - | - | AF223517.1 | - | - | AF116968.1 |
| [*Lapiedra martinezii*](https://www.ncbi.nlm.nih.gov/nuccore/AY751425.1) Lag. | 11 | - | - | - | - | - | AY751425.1 | HM011042.1 | AY434488.1 | - |
| [*Leptochiton quitoensis* (Herb.) Sealy](http://ccdb.tau.ac.il/Angiosperms/Amaryllidaceae/Leptochiton/Leptochiton%20quitoensis%20(Herb.)%20Sealy/) | 12 | - | - | - | - | - | AF223521.1 | - | FJ264225.1 | AF116970.1 |
| [*Leucojum aestivum*](https://www.ncbi.nlm.nih.gov/nuccore/AY751420.1) L. | 11 | 63.95 | * | - | - | - | AY751420.1 | AY101314.1 | AY747087.1 | KM360851.1 |
| [*Leucojum vernum*](https://www.ncbi.nlm.nih.gov/nuccore/AY101293.1) L. | 10 | 70.11 | * | - | - | - | AY101293.1 | AY101326.1 | JQ044761.1 | KF997480.1 |
| [*Lycoris albiflora*](https://www.ncbi.nlm.nih.gov/nuccore/FJ907413.1) Koidz. | 9 |  | - | 4 | 6 | Chang et al. 2009 | FJ907413.1 | AB243652.1 |  |  |
| [*Lycoris aurea*](https://www.ncbi.nlm.nih.gov/nuccore/AY942710.1) (L'Hér.) Herb. | 7 | 60.82 | Jiang et al. 2017 | 6 | 12 | Chang et al. 2009 |  |  | AY747091.1 |  |
| [*Lycoris chinensis*](https://www.ncbi.nlm.nih.gov/nuccore/AY942709.1) Traub | 8 | 64.84 | * | 10 | 12 | Chang et al. 2009 |  | KC704512.1 |  | KC704781.1 |
| *Lycoris koreana* Nakai | 11 | - | - | - | - | - | AY942722.1 | JF972929.1 | JX903349.1 | JF972895.1 |
| [*Lycoris haywardii*](https://www.ncbi.nlm.nih.gov/nuccore/AY942721.1)Traub | 11 | 47.92 | * | 3 | 4 | Chang et al. 2009 | AY942721.1 | AB243647.1 |  |  |
| *Lycoris longituba* Y.C.Hsu & G.J.Fan | 8 | 62.78 | * | - | - | - | AY942714.1 | AB243651.1 |  |  |
| [*Lycoris radiata*](https://www.ncbi.nlm.nih.gov/nuccore/JX464287.1) (L'Hér.) Herb. | 11 | 47 | Jiang et al. 2017 | 4 | 4 | Chang et al. 2009 | JX464287.1 | JX464564.1 | KC217373.1 | KC704782.1 |
| [*Lycoris rosea*](https://www.ncbi.nlm.nih.gov/nuccore/AY942720.1)Traub & Moldenke | 11 | - | - | 3 | 4 | Chang et al. 2009 | AY942720.1 | AB243648.1 |  |  |
| [*Lycoris sprengeri*](https://www.ncbi.nlm.nih.gov/nuccore/JX464288.1) Comes ex Baker | 11 | 48.72 | Jiang et al. 2017 | 2 | 4 | Chang et al. 2009 | JX464288.1 | JX464565.1 |  |  |
| [*Lycoris squamigera*](https://www.ncbi.nlm.nih.gov/nuccore/AY942730.1) Maxim. | 13 | - | - | - | - | - | AY942730.1 | AB243653.1 | AY747079.1 | KC704784.1 |
| [*Myostema elwesii*](https://www.ncbi.nlm.nih.gov/nuccore/FJ349199.1) (C.H.Wright) Ravenna | 18 | - | - | - | - | - | FJ349199.1 |  |  |  |
| *Narcissus abscissus* (Haw.) Schult. & Schult.f. | 7 | 26.4 | * | - | - | - |  | KR872970.1 | KT210952.1 |  |
| [*Narcissus assoanus* Dufour ex Schult. & Schult.](http://ccdb.tau.ac.il/Angiosperms/Amaryllidaceae/Narcissus/Narcissus%20assoanus%20Dufour%20ex%20Schult.%20&%20Schult./) | 7 | 18.8 | * | - | - | - | JX464290.1 | JX464567.1 |  |  |
| [*Narcissus asturiensis*](https://www.ncbi.nlm.nih.gov/nuccore/JX464291.1) (Jord.) Pugsley | 7 | 24.2 | * | - | - | - | JX464291.1 | JX464568.1 | U79205.1 |  |
| [*Narcissus atlanticus* Stern](http://ccdb.tau.ac.il/Angiosperms/Amaryllidaceae/Narcissus/Narcissus%20atlanticus%20Stern/) | 7 |  | * | - | - | - |  | KT033140.1 |  |  |
| *Narcissus broussonetii* Lag. | 11 | 37.4 | * | - | - | - |  | KR872998.1 | KT124411.1 |  |
| [*Narcissus bulbocodium*](https://www.ncbi.nlm.nih.gov/nuccore/JX464292.1) L. | 13 | 14.20 | * | - | - | - | JX464292.1 | JX464569.1 | U79193.1 |  |
| [*Narcissus calcicola*](https://www.ncbi.nlm.nih.gov/nuccore/JX464293.1) Mendonça | 7 | 26.6 | * | - | - | - | JX464293.1 | JX464570.1 | AY747093.1 |  |
| [*Narcissus  cantabricus*](https://www.ncbi.nlm.nih.gov/nuccore/KC607944.1) DC. | 7 | 14.5 | * | - | - | - | KC607944.1 |  | U79194.1 |  |
| [*Narcissus cavanillesii* Barra & G. López](http://ccdb.tau.ac.il/Angiosperms/Amaryllidaceae/Narcissus/Narcissus%20cavanillesii%20Barra%20&%20G.%20L%C3%B3pez/) | 14 | 31.57 | Marques et al. 2012 | - | - | - | KM211741.1 | KR872965.1 |  |  |
| [*Narcissus cernuus*](https://www.ncbi.nlm.nih.gov/nuccore/KC607960.1) Salisb. | 7 |  | - | - | - | - | KC607960.1 |  |  |  |
| [*Narcissus cuatrecasasii* F. Casas & M. Laínz & R. Rejón](http://ccdb.tau.ac.il/Angiosperms/Amaryllidaceae/Narcissus/Narcissus%20cuatrecasasii%20F.%20Casas%20&%20M.%20La%C3%ADnz%20&%20R.%20Rej%C3%B3n/) | 7 | 31.7 | * | - | - | - |  | KC608018.1 |  |  |
| *Narcissus cyclamineus* DC. | 7 | 26 | * | - | - | - |  | KT021575.1 | KT340525.1 |  |
| [*Narcissus dubius* Gouan](http://ccdb.tau.ac.il/Angiosperms/Amaryllidaceae/Narcissus/Narcissus%20dubius%20Gouan/) | 25 | 66.3 | * | - | - | - |  |  | U79215.1 |  |
| [*Narcissus elegans* (Haw.) Spach](http://ccdb.tau.ac.il/Angiosperms/Amaryllidaceae/Narcissus/Narcissus%20elegans%20(Haw.)%20Spach/) | 10 | 30.66 | Marques et al. 2012 | - | - | - |  |  |  | AF116972.1 |
| *Narcissus gaditanus* Boiss. & Reut. | 7 | 19.3 | * | - | - | - |  | KC608017.1 | U79201.1 |  |
| [*Narcissus hedraeanthus* (Webb & Heldr.) Colmeiro](http://ccdb.tau.ac.il/Angiosperms/Amaryllidaceae/Narcissus/Narcissus%20hedraeanthus%20(Webb%20&%20Heldr.)%20Colmeiro/) | 7 | 13.1 | * | - | - | - |  | KT021593.1 |  |  |
| [*Narcissus hispanicus*](https://www.ncbi.nlm.nih.gov/nuccore/FJ410299.1) Gouan | 7 | 25.8 | * | - | - | - | FJ410299.1 |  | U79206.1 |  |
| *Narcissus jacetanus* Fern.Casas | 7 | 22.3 | * | - | - | - |  | KT021600.1 | KT340546.1 |  |
| [*Narcissus jonquilla* L.](http://ccdb.tau.ac.il/Angiosperms/Amaryllidaceae/Narcissus/Narcissus%20jonquilla%20L./) | 7 | 32.8 | * | - | - | - |  | JX464571.1 |  |  |
| *Narcissus lusitanicus* Dorda & Fern.Casas | 7 | 16.9 | * | - | - | - |  | KT033112.1 | KT799100.1 |  |
| *Narcissus moleroi* Fern.Casas | 7 | 26.1 | * | - | - | - |  | KR872977.1 | KT210959.1 |  |
| *Narcissus miniatus* Donn.-Morg., Koop. & Zonn. | 15 | 53.1 | Marques et al. 2012 | - | - | - | KY971390.1 | KM349580.1 | HM372505.1 |  |
| *Narcissus nevadensis* Pugsley | 7 | 38.2 | * | - | - | - |  |  | AY357134.1 |  |
| [*Narcissus nivalis* Graells](http://ccdb.tau.ac.il/Angiosperms/Amaryllidaceae/Narcissus/Narcissus%20nivalis%20Graells/) | 7 |  |  | - | - | - |  | KT021634.1 |  |  |
| [*Narcissus obesus* Salisb*.*](http://ccdb.tau.ac.il/Angiosperms/Amaryllidaceae/Narcissus/Narcissus%20obesus%20Salisb./) | 13 | 26.5 | * | - | - | - |  | KT211011.1 |  |  |
| *Narcissus obsoletus* (Haw.) Spach | 15 | 43.85 | Pustahija et al. 2017 | - | - | - |  |  | JF973180.1 |  |
| [*Narcissus papyraceus* K. Gawl.](http://ccdb.tau.ac.il/Angiosperms/Amaryllidaceae/Narcissus/Narcissus%20papyraceus%20K.%20Gawl./) | 11 | 33.7 | * | - | - | - |  | HM011045.1 |  | HM850195.1 |
| *Narcissus poeticus* L. | 7 | 24.33 | * | - | - | - |  | KT033072.1 | KT124417.1 |  |
| [*Narcissus primigenius* (F. Suárez) F. Casas & Laínz](http://ccdb.tau.ac.il/Angiosperms/Amaryllidaceae/Narcissus/Narcissus%20primigenius%20(F.%20Su%C3%A1rez)%20F.%20Casas%20&%20La%C3%ADnz/) | 7 | 21.7 | * | - | - | - |  | KT033075.1 |  |  |
| *Narcissus pseudonarcissus* L. | 7 | 23.5 | * | - | - | - | AY751415.1 |  | AY747095.1 |  |
| [*Narcissus romieuxii* Braun-Blanq. & Maire](http://ccdb.tau.ac.il/Angiosperms/Amaryllidaceae/Narcissus/Narcissus%20romieuxii%20Braun-Blanq.%20&%20Maire/) | 14 | 28.8 | * | - | - | - |  | KT033085.1 |  |  |
| [*Narcissus rupicola*](https://www.ncbi.nlm.nih.gov/nuccore/JX464295.1) Dufour | 7 | 26.6 | * | - | - | - | JX464295.1 | JX464574.1 | U79191.1 |  |
| [*Narcissus scaberulus* Henriq*.*](http://ccdb.tau.ac.il/Angiosperms/Amaryllidaceae/Narcissus/Narcissus%20scaberulus%20Henriq./) | 7 | 26.3 | * | - | - | - |  | KT033096.1 |  |  |
| [*Narcissus serotinus* L.](http://ccdb.tau.ac.il/Angiosperms/Amaryllidaceae/Narcissus/Narcissus%20serotinus%20L./) | 15 | 43.85 | * | - | - | - | KM211772.1 | KM349592.1 |  |  |
| [*Narcissus tazetta* L.](http://ccdb.tau.ac.il/Angiosperms/Amaryllidaceae/Narcissus/Narcissus%20tazetta%20L./) | 11 | 30.3 | * | - | - | - |  | HM011047.1 |  | KC704785.1 |
| [*Narcissus triandrus* L.](http://ccdb.tau.ac.il/Angiosperms/Amaryllidaceae/Narcissus/Narcissus%20triandrus%20L./) | 7 | 19 | * | - | - | - | KC607968.1 | KC608016.1 | U79197.1 |  |
| [*Narcissus viridiflorus*](https://www.ncbi.nlm.nih.gov/nuccore/AY751416.1) Schousb. | 14 | 63.5 | * | - | - | - | AY751416.1 |  | AY747096.1 |  |
| *Nerine bowdenii* W.Watson | 11 | 35.3 | * | - | - | - | JX464271.1 | JX464535.1 | KM359737.1 | AF116973.1 |
| [*Nerine humilis*](http://www.ncbi.nlm.nih.gov/nuccore/JX464272.1) (Jacq.) Herb. | 11 | 25.3 | * | - | - | - | JX464272.1 | JX464536.1 |  |  |
| [*Nerine huttoniae*](http://www.ncbi.nlm.nih.gov/nuccore/JX464273.1) Schönland | 11 | - | - | - | - | - | JX464273.1 | JX464537.1 |  |  |
| [*Nerine laticoma*](http://www.ncbi.nlm.nih.gov/nuccore/JX464274.1) (Ker Gawl.) T.Durand & Schinz | 11 | 26.8 | * | - | - | - | JX464274.1 | JX464538.1 |  |  |
| [*Nerine masonorum*](http://www.ncbi.nlm.nih.gov/nuccore/JX464275.1) L.Bolus | 11 | 22.7 | * | - | - | - | JX464275.1 | JX464539.1 |  |  |
| [*Nerine platypetala*](http://www.ncbi.nlm.nih.gov/nuccore/JX464276.1) McNeil | 11 | 22.3 | * | - | - | - | JX464276.1 | JX464540.1 |  |  |
| [*Nerine undulata*](http://www.ncbi.nlm.nih.gov/nuccore/JX464277.1)(L.) Herb. | 11 | 28.4 | * | - | - | - | JX464277.1 | JX464541.1 |  |  |
| [*Pamianthe peruviana* Stapf](http://ccdb.tau.ac.il/Angiosperms/Amaryllidaceae/Pamianthe/Pamianthe%20peruviana%20Stapf/) | 23 | - | - | - | - | - | AF223546.1 |  |  | AF116974.1 |
| [*Pancratium canariense*](https://www.ncbi.nlm.nih.gov/nuccore/HM010979.1) Ker Gawl. | 11 | - | - | - | - | - | HM010979.1 | AY101344.1 | AY747097.1 | AF116975.1 |
| [*Pancratium maritimum*](https://www.ncbi.nlm.nih.gov/nuccore/HM010981.1) L. | 11 | 60.1 | * | - | - | - | HM010981.1 | HM011039.1 | HE565492.1 | HM850226.1 |
| [*Pancratium tenuifolium*](https://www.ncbi.nlm.nih.gov/nuccore/AF223537.1) Hochst. ex A.Rich. | 11 | - | - | - | - | - | AF223537.1 | FN594921.1 | AY747098.1 | JQ025067.1 |
| [*Pancratium trianthum*](https://www.ncbi.nlm.nih.gov/nuccore/HE565497.1) Herb. | 11 | - | - | - | - | - |  |  |  | HE565513.1 |
| [*Pancratium zeylanicum*](https://www.ncbi.nlm.nih.gov/nuccore/AY751431.1) L. | 11 | - | - | - | - | - | AY751431.1 |  |  | HE565515.1 |
| [*Paramongaia weberbaueri* Velarde](http://ccdb.tau.ac.il/Angiosperms/Amaryllidaceae/Paramongaia/Paramongaia%20weberbaueri%20Velarde/) | 23 | - | - | - | - | - | AF223536.1 | JX903571.1 | AY225040.1 | AF116976.1 |
| [*Phaedranassa dubia*](https://www.ncbi.nlm.nih.gov/nuccore/JX464326.1) (Kunth) J.F.Macbr. | 23 | - | - | - | - | - | JX464326.1 | FN663904.1 |  | AF116977.1 |
| [*Phycella australis* Ravenna](http://ccdb.tau.ac.il/Angiosperms/Amaryllidaceae/Phycella/Phycella%20australis%20Ravenna/) | 8 | - | - | - | - | - | KC207442.1 |  | KC217397.1 |  |
| [*Placea arzae* Phil*.*](http://ccdb.tau.ac.il/Angiosperms/Amaryllidaceae/Placea/Placea%20arzae%20Phil./) | 8 | - | - | - | - | - | FJ349191.1 |  | FJ264228.1 |  |
| [*Placea germainii* Phil*.*](http://ccdb.tau.ac.il/Angiosperms/Amaryllidaceae/Placea/Placea%20germainii%20Phil./) | 8 | - | - | - | - | - | KC207447.1 |  |  |  |
| [*Placea lutea* Phil*.*](http://ccdb.tau.ac.il/Angiosperms/Amaryllidaceae/Placea/Placea%20lutea%20Phil./) | 8 | - | - | - | - | - | KC207448.1 |  |  |  |
| [*Placea ornata* Miers](http://ccdb.tau.ac.il/Angiosperms/Amaryllidaceae/Placea/Placea%20ornata%20Miers/) | 8 | - | - | - | - | - | KC207449.1 |  |  |  |
| [*Plagiolirion horsmannii* Baker](http://ccdb.tau.ac.il/Angiosperms/Amaryllidaceae/Plagiolirion/Plagiolirion%20horsmannii%20Baker/) | 23 | - | - | - | - | - | FJ349193.1 |  |  |  |
| [*Rauhia multiflora*](https://www.ncbi.nlm.nih.gov/nuccore/AF223522.1) (Kunth) Ravenna | 23 | - | - | - | - | - | AF223522.1 | JX464605.1 |  |  |
| [*Rhodophiala advena*](https://www.ncbi.nlm.nih.gov/nuccore/KC207455.1)(Ker Gawl.) Traub | 9 | - | - | - | - | - | KC207455.1 |  | KC217410.1 |  |
| [*Rhodophiala araucana*](https://www.ncbi.nlm.nih.gov/nuccore/KC207459.1)(Phil.) Traub | 27 | - | - | - | - | - | KC207459.1 | JX464586.1 | KC217415.1 |  |
| [*Rhodophiala bagnoldii*](https://www.ncbi.nlm.nih.gov/nuccore/KC207460.1) (Herb.) Traub | 9 | - | - | - | - | - | KC207460.1 |  | FJ264230.1 |  |
| [*Rhodophiala chilensis*](https://www.ncbi.nlm.nih.gov/nuccore/JX464310.1) (L'Hér.) Traub | 9 | - | - | - | - | - | JX464310.1 | JX464588.1 |  |  |
| [*Rhodophiala montana*](https://www.ncbi.nlm.nih.gov/nuccore/KC207461.1) (Phil.) Traub | 9 | - | - | - | - | - | KC207461.1 |  | KC217417.1 |  |
| [*Rhodophiala splendens*](https://www.ncbi.nlm.nih.gov/nuccore/KC207463.1) (Renjifo) Traub | 9 | - | - | - | - | - | KC207463.1 |  | KC217419.1 |  |
| [*Rhodophiala tiltilensis*](https://www.ncbi.nlm.nih.gov/nuccore/KC207464.1) (Traub & Moldenke) Traub | 8 | - | - | - | - | - | KC207464.1 |  | KC217421.1 |  |
| [*Scadoxus multiflorus*](https://www.ncbi.nlm.nih.gov/nuccore/HM140812.1) (Martyn) Raf. | 9 | 88.4 | * | 4 | 3 | Monkheang et al. 2016 | HM140812.1 | JX464560.1 |  |  |
| [*Scadoxus puniceus*](https://www.ncbi.nlm.nih.gov/nuccore/HM140814.1) (L.) Friis & Nordal | 9 | - | - | - | - | - | HM140814.1 | JX464561.1 | JX903356.1 | AM234808.1 |
| [*Sprekelia formosissima*](https://www.ncbi.nlm.nih.gov/nuccore/JX464311.1) (L.) Herb. | 61 | 130.89 | * | - | - | - | JX464311.1 | JX464589.1 | AY434476.1 | AF116982.1 |
| [*Sternbergia candida* B. Mathew & T. Baytop](http://ccdb.tau.ac.il/Angiosperms/Amaryllidaceae/Sternbergia/Sternbergia%20candida%20B.%20Mathew%20&%20T.%20Baytop/) | 10 | - | - | - | - | - | HM010956.1 | HM011040.1 |  |  |
| [*Sternbergia clusiana* (K. Gawl.) K. Gawl. ex Spreng.](http://ccdb.tau.ac.il/Angiosperms/Amaryllidaceae/Sternbergia/Sternbergia%20clusiana%20(K.%20Gawl.)%20K.%20Gawl.%20ex%20Spreng./) | 10 | - | - | - | - | - | HM010961.1 | HM011034.1 |  |  |
| [*Sternbergia colchiciflora* Waldst. & Kit.](http://ccdb.tau.ac.il/Angiosperms/Amaryllidaceae/Sternbergia/Sternbergia%20colchiciflora%20Waldst.%20&%20Kit./) | 10 | 39..82 | * | - | - | - | HM010958.1 | HM011036.1 |  |  |
| [*Sternbergia pulchella* Boiss. & Blanche](http://ccdb.tau.ac.il/Angiosperms/Amaryllidaceae/Sternbergia/Sternbergia%20pulchella%20Boiss.%20&%20Blanche/) | 10 | - | - | - | - | - |  | HM011032.1 |  |  |
| [*Sternbergia vernalis*](http://ccdb.tau.ac.il/countsByMatchedName/Sternbergia%20fischeriana%20(Herb.)%20Roem.) (Mill.) Gorer & J.H.Harvey | 11 | - | - | - | - | - | HM010957.1 | HM011033.1 |  |  |
| [*Strumaria aestivalis*](http://www.ncbi.nlm.nih.gov/nuccore/AF373082.1) Snijman | 10 | - | - | - | - | - | AF373082.1 |  |  |  |
| [*Strumaria bidentata*](http://www.ncbi.nlm.nih.gov/nuccore/AF373081.1) Schinz | 10 | - | - | - | - | - | AF373081.1 |  |  |  |
| [*Strumaria chaplinii*](http://www.ncbi.nlm.nih.gov/nuccore/AF373085.1) (W.F.Barker) Snijman | 10 | - | - | - | - | - | AF373085.1 |  |  |  |
| [*Strumaria discifera*](http://www.ncbi.nlm.nih.gov/nuccore/JX464278.1) Marloth ex Snijman | 10 | - | - | - | - | - | JX464278.1 | JX464542.1 |  |  |
| [*Strumaria picta*](http://www.ncbi.nlm.nih.gov/nuccore/AF373099.1) W.F.Barker | 10 | - | - | - | - | - | AF373099.1 |  |  |  |
| [*Strumaria tenella*](http://www.ncbi.nlm.nih.gov/nuccore/AF373097.1) (L.f.) Snijman | 10 | - | - | - | - | - | AF373097.1 |  |  |  |
| [*Strumaria truncata*](http://www.ncbi.nlm.nih.gov/nuccore/JX464280.1) Jacq. | 10 | - | - | - | - | - | JX464280.1 | JX464544.1 |  | AF116985.1 |
| [*Strumaria watermeyeri*](http://www.ncbi.nlm.nih.gov/nuccore/AF373091.1) L.Bolus | 10 | - | - | - | - | - | AF373091.1 | AB017301.1 | FJ264236.1 |  |
| [*Traubia modesta* (Phil.) Ravenna](http://ccdb.tau.ac.il/Angiosperms/Amaryllidaceae/Traubia/Traubia%20modesta%20(Phil.)%20Ravenna/) | 8 | - | - | - | - | - | KC207468.1 |  | FJ264237.1 |  |
| [*Tristagma nivale* Poepp*.*](http://ccdb.tau.ac.il/Angiosperms/Amaryllidaceae/Tristagma/Tristagma%20nivale%20Poepp./) | 8 | - | - | - | - | - | HQ393024.1 | JX903552.1 | HQ392942.1 | JX903141.1 |
| [*Vagaria ollivieri* Maire](http://ccdb.tau.ac.il/Angiosperms/Amaryllidaceae/Vagaria/Vagaria%20ollivieri%20Maire/) | 11 | - | - | - | - | - | AY751426.1 | HM011046.1 | AY747078.1 |  |
| [*Worsleya procera*](http://ccdb.tau.ac.il/countsByMatchedName/Worsleya%20rayneri%20(Hook.)%20Traub%20&%20Moldenke) (Lem.) Traub | 21 | - | - | - | - | - | KC207420.1 |  | AY747105.1 | AF116989.1 |
| [*Zephyranthes albiella*](https://www.ncbi.nlm.nih.gov/nuccore/KC207469.1) Traub | 19 | - | - | - | - | - | KC207469.1 |  | KC217425.1 |  |
| [*Zephyranthes atamasco*](https://www.ncbi.nlm.nih.gov/nuccore/AF223474.1) *(*L.) Herb. | 6 | - | - | - | - | - | AF223474.1 |  | FJ264238.1 |  |
| [*Zephyranthes candida*](https://www.ncbi.nlm.nih.gov/nuccore/JX464313.1) (Lindl.) Herb. | 19 | 38 | * | - | - | - | JX464313.1 | JX464591.1 |  | KC704788.1 |
| [*Zephyranthes citrina*](https://www.ncbi.nlm.nih.gov/nuccore/AF223495.1) Baker | 24 | 31.5 | * | - | - | - | AF223495.1 |  | KC217430.1 |  |
| [*Zephyranthes drummondii*](https://www.ncbi.nlm.nih.gov/nuccore/AF223488.1) D.Don | 24 | - | - | - | - | - | AF223488.1 |  | KC217432.1 |  |
| [*Zephyranthes lindleyana*](https://www.ncbi.nlm.nih.gov/nuccore/JX464314.1) Herb. | 48 | - | - | - | - | - | JX464314.1 | JX464592.1 |  |  |
| [*Zephyranthes macrosiphon*](https://www.ncbi.nlm.nih.gov/nuccore/KC207475.1) Baker | 24 | - | - | - | - | - | KC207475.1 |  | KC217435.1 |  |
| [*Zephyranthes mesochloa*](https://www.ncbi.nlm.nih.gov/nuccore/AF223492.1) Herb. ex Lindl. | 13 | - | - | 2 | 4 | Santos et al. 2014 | AF223492.1 |  | FJ264243.1 |  |
| [*Zephyranthes minima*](https://www.ncbi.nlm.nih.gov/nuccore/JX464315.1) *Herb.* | 10 | - | - | - | - | - | JX464315.1 | JX464593.1 | FJ264244.1 |  |
| [*Zephyranthes puertoricensis*](https://www.ncbi.nlm.nih.gov/nuccore/KC207477.1)Traub | 13 | - | - | - | - | - | KC207477.1 |  | KC217438.1 |  |
| [*Zephyranthes rosea*](https://www.ncbi.nlm.nih.gov/nuccore/AF223487.1) Lindl. | 12 | - | - | - | - | - | AF223487.1 |  | FJ264245.1 |  |
| [*Zephyranthes simpsonii*](https://www.ncbi.nlm.nih.gov/nuccore/AF223472.1)Chapm. | 24 | - | - | - | - | - | AF223472.1 | JX903577.1 | KC217440.1 | JX903167.1 |
| [*Zephyranthes smallii*](https://www.ncbi.nlm.nih.gov/nuccore/KC207478.1) (Alexander) Traub | 35 | - | - | - | - | - | KC207478.1 |  | KC217441.1 |  |
| [*Zephyranthes treatiae*](https://www.ncbi.nlm.nih.gov/nuccore/KC207479.1) S.Watson | 12 | - | - | - | - | - | KC207479.1 |  | KC217442.1 |  |
