# Supplementary material for: Divide to Conquer: Evolutionary History of Allioideae Tribes (Amaryllidaceae) Is Linked to Distinct Trends of Karyotype Evolution
Source: Front Plant Sci. 2020 Apr 7;11:320. doi: 10.3389/fpls.2020.00320 (PMC7155398; doi:10.3389/fpls.2020.00320)
Supplement: TABLE S2 — Number of species sampled for BAMM analysis and percentage relative to the total number of species reported for tribes Tulbaghieae, Gilliesieae (divided in subtribes Leucocoryninae and Gilliesiinae) and Allieae (divided in the three Allium evolutionary lineages). [file Table_2.pdf]

Supplementary Table 2 – Number of species sampled for BAMM analysis and percentage relative to the total number of species reported for tribes Tulbaghieae, Gilliesieae (divided in subtribes Leucocoryninae and Gilliesiinae) and Allieae (divided in the three *Allium* evolutionary lineages)

**Taxa**	**Number of sampled species in BAMM analysis**	**% of sampled species for BAMM analysis**
Allieae		
Allium 1st evolutionary lineage	23	16%
Allium 2nd evolutionary lineage	55	30%
Allium 3rd evolutionary lineage	73	14%
Gilliesieae		
Gilliesiinae	6	27%
Leucocoryninae	30	45%
Tulbaghieae	3	14%
