# Supplementary material for: Divide to Conquer: Evolutionary History of Allioideae Tribes (Amaryllidaceae) Is Linked to Distinct Trends of Karyotype Evolution
Source: Front Plant Sci. 2020 Apr 7;11:320. doi: 10.3389/fpls.2020.00320 (PMC7155398; doi:10.3389/fpls.2020.00320)
Supplement: TABLE S3 — Results from the evolutionary model analysis for chromosome number and genome size with Log-Likelihood (Log-Lk) and AIC scores for each of the nine models. The analysis were repeated seven times for different combinations of taxa. Most likely models are highlighted in bold. [file Table_3.pdf]

Supplementary Table 3 – Results from the evolutionary model analysis for chromosome number and genome size with Log-Likelihood (Log-Lk) and AIC scores for each of the nine models. The analysis were repeated seven times for different combinations of taxa. Most likely models are highlighted in bold.

	**Chromosome number analysis**			**Genome size analysis**		
**Taxa**	**Model**	**Log-Lk**	**AIC**	**Model**	**Log-Lk**	**AIC**
**Allioideae**	Brownian Motion	-577.49	1158.99	Brownian Motion	-473.66	951.32
	**OU**	**-489.35**	**984.70**	OU	-448.79	903.59
	Early-Burst	-577.49	1160.99	Early-Burst	-473.66	953.32
	Trend	-565.74	1137.49	Trend	-466.27	938.54
	White	-491.29	986.57	White	-462.11	928.29
	Kappa	-516.95	1039.91	Kappa	-460.00	926;01
	Lambda	-485.61	977.23	**Lambda**	**-447.12**	**900.24**
	Delta	-555.90	1117.81	Delta	-460.74	927.47
**Allieae**	Brownian Motion	-464.78	933.56	Brownian Motion	-382.72	769.47
	**OU**	**-388.59**	**783.18**	OU	-359.99	725.99
	Early-Burst	-464.78	935.56	Early-Burst	-382.73	771.47
	Trend	-453.56	913.12	Trend	-375.38	756.75
	White	-390.08	784.16	White	-369.11	742.21
	Kappa	-417.22	840.43	Kappa	-367.25	740.50
	Lambda	-389.06	784.12	**Lambda**	**-354.11**	**714.21**
	Delta	-444.50	894.99	Delta	-370.12	746.24
***Allium* 1**	Brownian Motion	-83.53	171.06	Brownian Motion	-58.48	120.97
	OU	-67.27	140.54	OU	-53.62	113.23
	Early-Burst	-83.53	173.06	Early-Burst	-58.48	122.97
	Trend	-80.90	167.80	Trend	-57.09	120.18
	**White**	**-67.27**	**138.54**	**White**	**-54.19**	**112.38**
	Kappa	-71.55	149.09	Kappa	-56.53	119.05
	Lambda	-67.27	140.54	Lambda	-54.19	114.38
	Delta	-78.97	163.95	Delta	-56.18	118.37
***Allium* 2**	Brownian Motion	-126.05	256.11	Brownian Motion	-167.54	339.09
	OU	-108.64	223.28	OU	-148.12	302.24
	Early-Burst	-126.06	258.11	Early-Burst	-167.54	341.09
	Trend	-122.02	250.03	Trend	-163.39	332.77
	**White**	**-108.64**	**221.29**	**White**	**-148.12**	**300.24**
	Kappa	-120.13	246.26	Kappa	-154.82	315.64
	Lambda	-108.64	223.29	Lambda	-148.12	302.24
	Delta	-119.04	244.08	Delta	-160.26	326.51
***Allium* 3**	Brownian Motion	-221.71	447.42	Brownian Motion	-154.14	312.29
	**OU**	**-193.32**	**392.65**	OU	-145.58	297.16
	Early-Burst	-221.71	449.42	Early-Burst	-154.14	314.29
	Trend	-216.15	438.30	Trend	-151.08	308.16
	White	-193.52	393.04	**White**	**-146.90**	**287.80**
	Kappa	-208.73	423.46	Kappa	-151.62	309.24
	Lambda	-194.52	395.03	Lambda	-146.90	299.80
	Delta	-211.99	429.98	Delta	-149.10	304.20
**Gilliesieae**	Brownian Motion	-99.87	203.74	Brownian Motion	-82.31	168.63
	OU	-87.17	180.33	**OU**	**-80.06**	**166.12**
	Early-Burst	-99.87	205.74	Early-Burst	-82.31	170.63
	Trend	-96.87	199.75	Trend	-80.91	167.82
	White	-87.15	178.30	White	-82.57	169.15
	Kappa	-89.39	184.78	Kappa	-82.02	170.03
	**Lambda**	**-85.95**	**177.89**	Lambda	-80.82	167.64
	Delta	-94.75	195.50	Delta	-80.27	166.54
**Tulbaghieae**	Brownian Motion	528.91	-1053.82	Brownian Motion	-	-
	OU	555.43	-1104.85	OU	-	-
	Early-Burst	557.70	-1109.39	Early-Burst	-	-
	Trend	555.40	-1104.80	Trend	-	-
	White	553.98	-1103.95	White	-	-
	Kappa	555.40	-1104.80	Kappa	-	-
	Lambda	555.40	-1104.80	Lambda	-	-
	**Delta**	**573.42**	**-1140.84**	Delta	-	-
